# Supplementary material for: Global patterns and trends of carbon monoxide poisoning: A comprehensive spatiotemporal analysis using joinpoint regression and ARIMA modeling, 1990–2021
Source: PLoS One. 2025 Aug 22;20(8):e0330778. doi: 10.1371/journal.pone.0330778 (PMC12373207; doi:10.1371/journal.pone.0330778)
Supplement: Attachment — Submitted filename: The Approval DocumentNO.2 (Approval No. P202309-1)English.pdf [file pone.0330778.s005.pdf]

Ethics Committee Approval Letter of Biomedical Research Involving Humans

Approval NO: P20230902-1-2
Study Title: Clinical Study on the Effect of Early Life and Medical Interventions on Reducing the Incidence and Severity of Carbon Monoxide Poisoning
Sponsor/Funder: Huzhou Municipal Science and Technology Bureau
Acceptance Number: P20230902-1
Principal Investigator: Weiguang Wang. **Responsible Department:** Emergency Department
Category of Review: Research Project. **Type of Review:** Expedited Review
Date of Review: 20240801 **Location of Review:** Ethics Committee Office
Items Reviewed: Research Progress Report
Evaluation: Approved
Decision: The committee's decision on this project is: ☑ Approval
Chair Signature: Xiaozheng Wang
Date of Issue: 2024.8.1
Stamp of EC: Ethics Committee Seal
Contact Information: Phone: 0572-5665621 Email: ajxrmyy@163.com
Address: No. 699, Tianhuangping Middle Road, Changshuo Street, Anji County, Huzhou City, Zhejiang Province。
Period of Validity: From the date of initial approval by this ethics committee, the clinical research should be initiated in this hospital. If not initiated within the deadline, this approval will be automatically invalidated.
Continue Review: The review frequency is once every 12 months from the date of approval. The first report should be submitted one month before August 1, 2025.The Ethics Committee has the right to change the review frequency according to the actual progress.
Statement: Declaration: The responsibilities, personnel composition, operating procedures, and records of The Ethics Committee comply with the "Regulations on Ethical Review of Biomedical Research Involving Humans," "Regulations on Ethical Review of Life Sciences and Medical Research Involving Humans," "International Ethical Guidelines for Health-Related Research Involving Humans," the Declaration of Helsinki, GCP, and ICH-GCP, as well as other relevant domestic laws and regulations.

Notice:

1. Please follow the ethical principles in the relevant laws, regulations, and rules of our country.
2. Please conduct this research in accordance with the clinical research plan, informed consent form, recruitment materials, etc., approved by The Ethics Committee, and protect the health and rights of research participants. Any modifications to the research plan, informed consent form, and recruitment materials must be reviewed and approved by The Ethics Committee before implementation.
3. Any SAE/SUSAR occurring in this hospital and safety updates during the research and development period must be submitted to The Ethics Committee in a timely manner according to the latest requirements of NMPA/GCP. SAE/SUSAR occurring in other domestic and foreign centers need to be regularly summarized and evaluated before being submitted to The Ethics Committee.
4. Based on the report, The Ethics Committee has the right to make new decisions on its assessment.
5. From today onwards, regardless of whether the research has started, please submit a research progress report one month before the tracking review date expires.
6. The sponsor should submit a summary of the research progress report to the leading unit's ethics committee; when any situation that may significantly affect the progress of the research or increase the risk to participants occurs, the applicant should submit a written report toThe Ethics committee in a timely manner.
7. If the research includes participants who do not meet the inclusion criteria or meet the exclusion criteria, participants who should have been withdrawn from the study according to the regulations but were not, participants who received incorrect treatment or dosage, or participants who received prohibited combined medications that did not comply with the research plan; or if there is any situation that may adversely affect the rights or health of the participants and the scientific nature of the research, violating GCP principles, the sponsor, monitor, or researcher should submit a non-compliance report.
8. If the applicant suspends or terminates the clinical research, please submit a suspension or termination report in a timely manner.
9. Upon completion of the clinical research, the applicant should submit a final report.
10. The collection, preservation, use, and provision of human genetic resources in our country should comply with the Regulations on the Administration of Human Genetic Resources of the People's Republic of China.
11. Before the implementation of any research project approved by The Ethics Committee, the applicant should register the relevant information of the research project on the clinical research registration and filing information system platform of

the National Health Commission, Drug Review Center, etc., in accordance with relevant regulations.
