# Supplementary material for: Global patterns and trends of carbon monoxide poisoning: A comprehensive spatiotemporal analysis using joinpoint regression and ARIMA modeling, 1990–2021
Source: PLoS One. 2025 Aug 22;20(8):e0330778. doi: 10.1371/journal.pone.0330778 (PMC12373207; doi:10.1371/journal.pone.0330778)
Supplement: Attachment — Submitted filename: PONE-D-25-08215_reviewed.pdf [file pone.0330778.s003.pdf]

PLOS ONE

Global Patterns and Trends of Carbon Monoxide Poisoning: A Systematic Analysis from 1990 to 2021

--Manuscript Draft--

**Manuscript Number:**	PONE-D-25-08215
**Article Type:**	Research Article
**Full Title:**	Global Patterns and Trends of Carbon Monoxide Poisoning: A Systematic Analysis from 1990 to 2021
**Short Title:**	Systematic Analysis for Global Patterns and Trends of Carbon Monoxide Poisoning
**Corresponding Author:**	Weiguang Wang<br>People's hospital of AnJi<br>AnJi City, CHINA
**Keywords:**	carbon monoxide poisoning; Global Burden of Disease; epidemiology; Socio-demographic Index; health disparities; temporal trends
**Abstract:**	背景<br><br>Carbon monoxide (CO) poisoning causes approximately 41,000 deaths annually worldwide. Despite its preventable nature, comprehensive analyses of global patterns and trends remain limited, particularly regarding its relationship with socioeconomic development.<br><br>Methods<br><br>Using data from the Global Burden of Disease Study 2021, we analyzed CO poisoning burden across 204 countries using joinpoint regression, spatial statistics, and ARIMA modeling. We examined age-standardized rates of incidence, mortality, and disability-adjusted life years (DALYs), and their association with the socio-demographic index (SDI).<br><br>Results<br><br>Global age-standardized incidence rates of CO poisoning decreased by 35.1% from 1990 (12.13 per 100,000) to 2021 (7.87 per 100,000). Eastern Europe showed the highest burden (37.98 per 100,000 in 2021). High-SDI regions demonstrated higher incidence but lower mortality rates compared to low-SDI regions. Males experienced 2.5 times higher mortality rates than females. SDI analysis revealed peak disease burdens at moderate development levels (SDI: 0.6-0.7).<br><br>Conclusions<br><br>While global CO poisoning burden has decreased significantly, marked regional disparities persist. Our findings suggest the need for differentiated interventions: upgrading heating infrastructure in high-burden regions while strengthening surveillance and treatment capabilities in low-resource settings. Future research should focus on identifying cost-effective prevention strategies and establishing comprehensive surveillance systems in developing regions.
**Order of Authors:**	Weiguang Wang
	Yongai Ling
	Xianwei Xiong
	Jiajie Zhou
**Additional Information:**	
**Question**	**Response**
**Financial Disclosure**	The author(s) received no specific funding for this work.

Powered by Editorial Manager® and ProduXion Manager® from Aries Systems Corporation

Enter a financial disclosure statement that describes the sources of funding for the work included in this submission. Review the submission guidelines for detailed requirements. View published research articles from *PLOS ONE* for specific examples.<br><br>This statement is required for submission and **will appear in the published article** if the submission is accepted. Please make sure it is accurate.<br><br>**Funded studies**<br>Enter a statement with the following details:<br>• Initials of the authors who received each award<br>• Grant numbers awarded to each author<br>• The full name of each funder<br>• URL of each funder website<br>• Did the sponsors or funders play any role in the study design, data collection and analysis, decision to publish, or preparation of the manuscript?<br><br>Did you receive funding for this work?	
**Competing Interests**<br><br>Use the instructions below to enter a competing interest statement for this submission. On behalf of all authors, disclose any competing interests that could be perceived to bias this work—acknowledging all financial support and any other relevant financial or non-financial competing interests.<br><br>This statement is required for submission and **will appear in the published article** if the submission is accepted. Please make sure it is accurate and that any funding sources listed in your Funding Information later in the submission form are also declared in your Financial Disclosure statement.	The authors have declared that no competing interests exist

View published research articles from *PLOS ONE* for specific examples.<br><br>**NO authors have competing interests**<br><br>Enter: *The authors have declared that no competing interests exist.*<br><br>**Authors with competing interests**<br><br>Enter competing interest details beginning with this statement:<br><br>*I have read the journal's policy and the authors of this manuscript have the following competing interests: [insert competing interests here]*<br><br>* typeset	
**Ethics Statement**<br><br>Enter an ethics statement for this submission. This statement is required if the study involved:<br><br>• Human participants<br>• Human specimens or tissue<br>• Vertebrate animals or cephalopods<br>• Vertebrate embryos or tissues<br>• Field research<br><br>Write "N/A" if the submission does not require an ethics statement.<br><br>General guidance is provided below. Consult the submission guidelines for detailed instructions. **Make sure that all information entered here is included in the Methods section of the manuscript.**	Ethics Approval No. (P202309-1),People's Hospital of Anji

**Format for specific study types**<br><br>**Human Subject Research (involving human participants and/or tissue)**<br>• Give the name of the institutional review board or ethics committee that approved the study<br>• Include the approval number and/or a statement indicating approval of this research<br>• Indicate the form of consent obtained (written/oral) or the reason that consent was not obtained (e.g. the data were analyzed anonymously)<br><br>**Animal Research (involving vertebrate animals, embryos or tissues)**<br>• Provide the name of the Institutional Animal Care and Use Committee (IACUC) or other relevant ethics board that reviewed the study protocol, and indicate whether they approved this research or granted a formal waiver of ethical approval<br>• Include an approval number if one was obtained<br>• If the study involved *non-human primates*, add *additional details* about animal welfare and steps taken to ameliorate suffering<br>• If anesthesia, euthanasia, or any kind of animal sacrifice is part of the study, include briefly which substances and/or methods were applied<br><br>**Field Research**<br><br>Include the following details if this study involves the collection of plant, animal, or other materials from a natural setting:<br>• Field permit number<br>• Name of the institution or relevant body that granted permission	
**Data Availability**<br><br>Authors are required to make all data underlying the findings described fully available, without restriction, and from the time of publication. PLOS allows rare exceptions to address legal and ethical concerns. See the PLOS Data Policy and FAQ for detailed information.	Yes - all data are fully available without restriction

Powered by Editorial Manager® and ProduXion Manager® from Aries Systems Corporation

A Data Availability Statement describing where the data can be found is required at submission. Your answers to this question constitute the Data Availability Statement and **will be published in the article**, if accepted.<br><br>**Important:** Stating 'data available on request from the author' is not sufficient. If your data are only available upon request, select 'No' for the first question and explain your exceptional situation in the text box.<br><br>Do the authors confirm that all data underlying the findings described in their manuscript are fully available without restriction?	
**Describe where the data may be found in full sentences. If you are copying our sample text, replace any instances of XXX with the appropriate details.**<br><br>• If the data are **held or will be held in a public repository**, include URLs, accession numbers or DOIs. If this information will only be available after acceptance, indicate this by ticking the box below. For example: *All XXX files are available from the XXX database (accession number(s) XXX, XXX.).*<br>• If the data are all contained **within the manuscript and/or Supporting Information files**, enter the following: *All relevant data are within the manuscript and its Supporting Information files.*<br>• If neither of these applies but you are able to provide **details of access elsewhere**, with or without limitations, please do so. For example:<br><br>*Data cannot be shared publicly because of [XXX]. Data are available from the XXX Institutional Data Access / Ethics Committee (contact via XXX) for researchers who meet the criteria for access to confidential data.*<br><br>*The data underlying the results presented in the study are available from (include the name of the third party*	All relevant data are within the manuscript and its Supporting Information files

and contact information or URL).<br>• This text is appropriate if the data are owned by a third party and authors do not have permission to share the data.<br><br>* typeset	
Additional data availability information:	

Powered by Editorial Manager® and ProduXion Manager® from Aries Systems Corporation

Manuscript Modified
Click here to access/download;Manuscript;Global Patterns and Trends of Carbon Monoxide Poisoning C.doc

Global Patterns and Trends of Carbon Monoxide Poisoning: A Systematic Analysis from 1990 to 2021

Weiguang Wang[1] , Yongai Ling[1], Xianwei Xiong[1], Jiajie Zhou[2]

Weiguang Wang is the corresponding author and first author

Email:batteryweiger2018@163.com

[1] Emergency Department,People's Hospital of Anji,An'ji(City),Zhejiang Province,China(Country).
[2] Department of Gastrointestinal Surgery, The Affiliated Huaian No.1 People's Hospital of Nanjing Medical University, Huai'an (City), Jiangsu Province, China (Country)

Abstract

Background: Carbon monoxide (CO) poisoning causes approximately 41,000 deaths annually worldwide. Despite its preventable nature, comprehensive analyses of global patterns and trends remain limited, particularly regarding its relationship with socioeconomic development.

Methods: Using data that were accessed on 1/1/2025 from the Global Burden of Disease Study 2021, we analyzed CO poisoning burden across 204 countries using joinpoint regression, spatial statistics, and ARIMA modeling. We examined age-standardized rates of incidence, mortality, and disability-adjusted life years (DALYs), and their association with the socio-demographic index (SDI).

Results: Global age-standardized incidence rates of CO poisoning decreased by 35.1% from 1990 (12.13 per 100,000) to 2021 (7.87 per 100,000). Eastern Europe showed the highest burden (37.98 per 100,000 in 2021). High-SDI regions demonstrated higher incidence but lower mortality rates compared to low-SDI regions. Males experienced 2.5 times higher mortality rates than females. SDI analysis revealed peak disease burdens at moderate development levels (SDI: 0.6-0.7).

Conclusions: While global CO poisoning burden has decreased significantly, marked regional disparities persist. Our findings suggest the need for differentiated interventions: upgrading heating infrastructure in high-burden regions while strengthening surveillance and treatment capabilities in low-resource settings. Future research should focus on identifying cost-effective prevention strategies and establishing comprehensive surveillance systems in developing regions.

Keywords: carbon monoxide poisoning; global burden of disease; epidemiology; socio-demographic index; health disparities; temporal trends

Introduction

Carbon monoxide (CO) poisoning is one of the most common causes of fatal poisoning worldwide[1]. The burden of CO poisoning remains significant despite its preventable nature. Previous research has estimated that approximately 970,000 poisoning incidents occur annually worldwide, resulting in around 41,000 deaths[2]. CO is a colorless, odorless, and tasteless toxic gas produced by the incomplete combustion of carbon-containing fuels. Common sources include faulty heating systems, poorly ventilated cooking appliances, vehicle exhaust, and the burning of charcoal or other fuels in enclosed spaces[3].

The health impacts of CO poisoning can be severe and far-reaching. Acute exposure can lead to symptoms ranging from mild (headache, dizziness, nausea) to severe (loss of consciousness, heart damage, and death)[4]. Long-term sequelae among survivors may include neurological deficits, cognitive impairment, and cardiovascular complications. The economic burden is also substantial, encompassing direct medical costs, lost productivity, and long-term care expenses[5].

Several studies[6-8] have attempted to characterize the burden of CO poisoning at various levels. Long[6] et al. examined global mortality trends from 1990-2017, while Cui[7] et al. focused specifically on China's burden from 1990-2019. The Lancet Public Health[8] provided insights into mortality patterns from 2000-2021. However, existing research has several important limitations.

First, previous studies[6-8] primarily focused on mortality rates alone or were limited to specific regions like China. There is a lack of comprehensive analysis incorporating incidence, mortality, and disability-adjusted life years (DALYs) at the global level. This multi-dimensional understanding is crucial for fully grasping the public health impact of CO poisoning.

Second, although some studies[6-8] have examined regional variations in CO poisoning burden, they failed to systematically analyze the relationship between socio-demographic index (SDI) and CO poisoning burden at both regional and national levels. Understanding this relationship is crucial for policy-making and resource allocation, particularly in identifying vulnerable populations and regions.

Third, while existing studies[6-8] have described historical trends, there is no long-term prediction model for CO poisoning burden. Such predictions are essential for future healthcare planning and prevention strategies, especially given changing global patterns of energy use and climate.

Fourth, although gender disparities in CO poisoning have been noted in previous studies[6-8], systematic analysis of sex-specific patterns in incidence, mortality and DALYs across different regions and time periods is lacking. Understanding these patterns is vital for developing targeted prevention strategies.

Additionally, most existing studies[6-8] are descriptive in nature and do not provide in-depth analysis of geographical gradients or explore the complex factors contributing to regional variations in CO poisoning burden. Such detailed geographical analysis could reveal important patterns in disease burden distribution and help identify region-specific risk factors.

To address these significant gaps in knowledge, we conducted a comprehensive global analysis of CO poisoning burden using data from the Global Burden of Disease (GBD) Study 1990-2021. Our study aims were to: (1) quantify the global burden of CO poisoning across multiple dimensions including incidence, mortality, and disability-adjusted life years (DALYs); (2) analyze the relationship between socio-demographic development and CO poisoning burden at global, regional, and national levels; (3) develop predictive models estimating the future CO poisoning burden through 2050; (4) examine detailed geographical and sex-specific patterns in disease burden distribution; (5) explore the implications of these patterns for public health policy and prevention strategies.

This comprehensive analysis provides crucial insights for informing evidence-based policies and interventions to reduce CO poisoning burden worldwide. Understanding these patterns and trends is essential for developing targeted prevention strategies and allocating resources effectively to reduce the preventable burden of CO poisoning.

Methods

Study Design and Data Sources

We conducted a comprehensive analysis of acute carbon monoxide poisoning using the Global Burden of Disease (GBD) 2021 database, accessed through the Global Health Data Exchange (GHDx) platform[9]. The analysis covered the period from 1990 to 2021, encompassing data from 204 countries and territories. Our research framework adhered to the GATHER guidelines for health estimates reporting[10]. The data were accessed for research purposes on March 15, 2021. Ethical approval for the use of these data was obtained from the institutional review board (IRB) of People's Hospital of Anji,and the need for informed consent was waived due to the retrospective and anonymized nature of the data. All analyses were conducted in compliance with PLOS ONE's ethical guidelines for human subjects research.

Analytical Framework

The geographical distribution of disease burden was evaluated using spatial statistics, incorporating both Global Moran's I for overall clustering assessment and LISA analysis for identifying regional patterns[11]. We examined the relationship between disease burden and development level by analyzing health metrics against the Socio-demographic Index (SDI), with nations grouped into SDI quintiles.

Temporal trend analysis employed joinpoint regression methodology, utilizing both APC and AAPC metrics[12]. The analysis parameters allowed for a maximum of three joinpoints, with optimal model selection guided by Monte Carlo permutation testing. Long-term projections extending to 2050 were generated using a combination of ARIMA modeling and exponential smoothing techniques[13], with model selection based on AIC values and validated through rolling-origin cross-validation.

Burden Metrics

We assessed disease burden through multiple indicators, primarily focusing on DALYs (calculated as the sum of YLLs and YLDs), along with incidence, prevalence, and mortality rates[14]. To ensure comparability across regions and time periods, all rates were age-standardized using the WHO standard population. Statistical uncertainty was addressed through 95% uncertainty intervals, with significance determined at $p < 0.05$.

Computational Tools

Analyses were performed using a combination of statistical software packages: R 4.1.0 and Stata 16 for primary analyses, the National Cancer Institute's Joinpoint Regression Program (version 4.9.1.0) for trend assessment, and ArcGIS Pro/QGIS 3.16 for spatial visualization.

RESULTS

1.Epidemiological Patterns and Trends of Acute Carbon Monoxide Poisoning, 1990-2021

1.1 Global Burden and Temporal Trends (Table 1)

In 2021, there were 294,173.15 (95% UI: 209,197.11-395,043.48) cases of acute carbon monoxide poisoning globally, with an age-standardized incidence rate (ASR) of 7.87 (95% UI: 5.54-10.81) per 100,000 population. This represents a substantial reduction from 1990, when there were 693,139.82 (95% UI: 469,815.01-981,111.48) cases and an ASR of 12.13 (95% UI: 8.30-17.00) per 100,000 population. The estimated annual percentage change between 1990 and 2021 was -1.16% (95% UI: -1.35% to -0.96%).

Sex-specific patterns showed that in 1990, females had a higher ASR (12.92 per 100,000; 95% UI: 8.69-18.19) than males (11.35 per 100,000; 95% UI: 7.88-15.56). By 2021, both sexes showed decreased rates, with females at 8.16 (95% UI: 5.58-11.48) and males at 7.60 (95% UI: 5.46-10.26) per 100,000 population. The annual percentage change was -1.26% (95% UI: -1.44% to -1.07%) for females and -1.04% (95% UI: -1.24% to -0.84%) for males.

Across SDI quintiles, high-SDI regions recorded the highest ASR in 1990 at 24.77 (95% UI: 16.00-35.74) per 100,000, decreasing to 17.77 (95% UI: 11.48-25.96) in 2021. High-middle SDI regions showed similar patterns, with ASRs of 23.58 (95% UI: 16.66-31.97) in 1990 and 17.85 (95% UI: 12.82-24.28) in 2021. Low-SDI regions maintained the lowest rates throughout the study period, with ASRs of 3.39 (95% UI: 2.13-4.96) in 1990 and 2.78 (95% UI: 1.87-4.00) in 2021.

In the GBD regional analysis, Eastern Europe showed the highest ASR in 1990 (55.71 per 100,000; 95% UI: 41.19-72.97), followed by High-income Asia Pacific (32.77 per 100,000; 95% UI: 21.77-46.85). By 2021, Eastern Europe's ASR had decreased to 37.98 (95% UI: 28.51-49.23). East Asia showed an increase in ASR from 11.17 (95% UI: 7.70-15.68) to 12.87 (95% UI: 9.26-17.06), with an annual percentage change of 0.97% (95% UI: 0.71-1.24%). Southern Latin America also demonstrated an increase, with an annual percentage change of 0.46% (95% UI: 0.19-0.72%).

1.2 Deaths and Mortality Trends (Table 2)

Global deaths from acute carbon monoxide poisoning decreased from 36,816.05 (95% UI: 31,520.91-44,285.07) in 1990 to 28,946.78 (95% UI: 19,894.89-33,510.18) in 2021. The age-standardized death rate (ASR) showed a more pronounced decline, from 0.76 (95% UI: 0.66-0.91) to 0.35 (95% UI: 0.24-0.40) per 100,000 population, with an estimated annual percentage change of -2.79% (95% UI: -3.14% to -2.44%).

Sex-stratified analysis revealed consistently higher mortality rates among males. In 1990, the male ASR was 1.07 (95% UI: 0.88-1.39) compared to 0.46 (95% UI: 0.32-0.63) for females per 100,000 population. By 2021, these rates had decreased to 0.50 (95% UI: 0.36-0.63) for males and 0.20 (95% UI: 0.10-0.25) for females, with similar annual percentage changes of -2.756% (95% UI: -3.12% to -2.39%) and -2.771% (95% UI: -3.09% to -2.46%), respectively.

Among SDI quintiles, high-middle SDI regions recorded the highest ASR in 1990 at 1.79 (95% UI: 1.66-2.05) per 100,000, decreasing to 0.76 (95% UI: 0.59-0.86) in 2021. Low-middle SDI regions maintained the lowest rates, declining from 0.20 (95% UI: 0.11-0.27) to 0.12 (95% UI: 0.07-0.16) per 100,000 population.

Geographically, Eastern Europe showed the highest mortality burden, with an ASR of 4.44 (95% UI: 4.33-4.52) per 100,000 in 1990, decreasing to 2.10 (95% UI: 1.95-2.25) in 2021. Central Europe experienced the steepest decline with an annual percentage change of -5.15% (95% UI: -5.42% to -4.89%). Notably, some regions showed increasing trends, including High-income North America (1.16%; 95% UI: 0.88-1.45%) and Andean Latin America (2.42%; 95% UI: 1.83-3.02%). East Asia maintained a substantial burden throughout the study period, with death numbers of 14,111.63 (95% UI: 10,243.22-21,401.62) in 1990 and 13,588.38 (95% UI: 6,818.19-17,624.96) in 2021.

1.3 Disability-Adjusted Life Years (Table 3)

The global burden of acute carbon monoxide poisoning measured in disability-adjusted life years (DALYs) decreased substantially from 1,991,539.89 (95% UI: 1,664,186.66-2,374,749.90) in 1990 to 1,228,861.20 (95% UI: 867,811.13-1,414,898.57) in 2021. The age-standardized DALY rate showed a marked reduction from 37.59 (95% UI: 31.75-44.76) to 15.22 (95% UI: 10.67-17.57) per 100,000 population, with an estimated annual percentage change of -3.18% (95% UI: -3.51% to -2.84%).

The sex-specific burden demonstrated pronounced disparities. Males experienced higher DALY rates, with an ASR of 51.84 (95% UI: 41.59-64.96) per 100,000 in 1990, decreasing to 21.52 (95% UI: 15.32-26.87) in 2021. Female rates declined from 23.13 (95% UI: 15.24-31.26) to 8.94 (95% UI: 4.58-10.63) per 100,000, with annual percentage changes of -3.13% (95% UI: -3.49% to -2.77%) for males and -3.23% (95% UI: -3.53% to -2.93%) for females.

Across SDI quintiles, high-middle SDI regions showed the highest burden with an ASR of 87.55 (95% UI: 80.24-100.93) per 100,000 in 1990, declining to 33.83 (95% UI: 26.92-38.06) in 2021. Low-middle SDI regions maintained the lowest rates, decreasing from 10.69 (95% UI: 5.41-15.27) to 6.23 (95% UI: 3.53-8.53) per 100,000 population.

In the regional analysis, Eastern Europe recorded the highest ASR in 1990 at 212.93 (95% UI: 207.51-217.50) per 100,000, dropping to 95.02 (95% UI: 88.66-101.06) in 2021. Central Europe showed the steepest decline with an annual percentage change of -5.40% (95% UI: -5.67% to -5.14%). Conversely, several regions exhibited increasing trends, including Andean Latin America (1.91%; 95% UI: 1.35-2.48%) and High-income North America (1.02%; 95% UI: 0.70-1.33%). East Asia demonstrated a substantial reduction in ASR from 67.98 (95% UI: 49.48-100.37) to 34.61 (95% UI: 17.91-43.44) per 100,000 population.

2. Global Geographic Distribution

2.1 Geographic Distribution of Age-Standardized Incidence Rates

The age-standardized incidence rates (ASR) of acute carbon monoxide poisoning showed marked geographic variation across the global regions (Figure 1). The highest rates were observed in the Balkan Peninsula and Northern Europe, with ASRs ranging from 26.83 to 41.60 per 100,000 population. The second-highest burden was found in North America and parts of Northern Asia, where ASRs ranged from 17.66 to 26.83 per 100,000 population.

Australia and parts of Central America displayed moderate incidence rates, falling within the range of 9.19 to 14.80 per 100,000 population. The Persian Gulf region and parts of Central Asia showed rates between 4.30 and 7.26 per 100,000 population.

Lower incidence rates were predominantly observed in Africa and Southeast Asia, where most regions recorded ASRs between 1.82 and 3.34 per 100,000 population. Specifically, Southeast Asian countries demonstrated rates in the lowest bracket (1.82-2.52 per 100,000 population), while most of sub-Saharan Africa showed rates between 2.52 and 3.34 per 100,000 population.

A clear gradient was visible from north to south in the Americas, with North America showing substantially higher rates (17.66-26.83 per 100,000) compared to South America, where rates varied but generally remained between 3.34 and 4.30 per 100,000 population, except for the southernmost regions which displayed higher rates.

2.2 Geographic Distribution of Age-Standardized Mortality Rates

The age-standardized mortality rates (ASR) for acute carbon monoxide poisoning exhibited distinct geographical patterns across the world (Figure 2). The highest mortality rates were concentrated in Northern Asia and parts of Eastern Europe, where ASRs reached between 0.63 and 2.72 per 100,000 population. The Balkan Peninsula region also demonstrated notably high mortality rates, falling within the range of 0.32 to 0.63 per 100,000 population.

The Persian Gulf region displayed elevated mortality rates, with ASRs ranging from 0.21 to 0.32 per 100,000 population. North America showed moderate mortality rates, with ASRs between 0.15 and 0.21 per 100,000 population. Similar levels were observed in parts of Central Asia and Northern Europe.

Lower mortality rates were documented across most of Southeast Asia and Africa, where ASRs generally ranged from 0.03 to 0.07 per 100,000 population. The lowest mortality rates (0.00-0.03 per 100,000 population) were observed in parts of South America, particularly in Brazil and surrounding regions.

A notable gradient was apparent in the Americas, with higher rates in North America (0.15-0.21 per 100,000) contrasting with lower rates in central regions, though Southern Latin America showed elevated rates comparable to those in North America. The mortality pattern in Europe displayed considerable heterogeneity, with a clear east-to-west gradient showing higher rates in Eastern Europe and lower rates in Western European countries.

2.3 Geographic Distribution of Age-Standardized DALY Rates

The age-standardized DALY rates for acute carbon monoxide poisoning showed substantial geographic variation worldwide (Figure 3). Northern Asia demonstrated

the highest burden, with rates ranging from 30.55 to 120.20 DALYs per 100,000 population. The Balkan Peninsula and parts of Eastern Europe also exhibited notably high rates, falling within the range of 16.75 to 30.55 DALYs per 100,000 population.

The Persian Gulf region and North America showed moderately high DALY rates, ranging from 10.58 to 16.75 per 100,000 population. Central Asian countries displayed intermediate burden levels, with rates typically between 8.09 and 10.58 DALYs per 100,000 population.

Lower DALY rates were observed across most of Africa and Southeast Asia, where rates generally ranged from 2.53 to 4.30 per 100,000 population. The lowest burden was documented in parts of South America, particularly Brazil, with rates between 0.87 and 2.05 DALYs per 100,000 population.

The Americas showed a clear north-south divide in DALY rates, with North America experiencing higher rates (10.58-16.75 per 100,000) compared to Central America (5.46-8.09 per 100,000), though Southern Latin America demonstrated elevated rates similar to North American levels. European DALY rates exhibited marked regional variation, with a pronounced east-to-west gradient showing substantially higher rates in Eastern Europe compared to Western European nations.

3 Global Analysis of the Relationship Between Socio-Demographic Index (SDI) and Carbon Monoxide Poisoning Disease Burden

3.1 Regional-Level Analysis

3.1.1 Regional Association Between SDI and Age-Standardized Incidence Rates

The age-standardized incidence rates (ASR) of acute carbon monoxide poisoning showed a strong positive correlation with socio-demographic index (SDI) across 21 global regions (Spearman's $r = 0.7611$, $p = 2.316e-142$) (Figure 4). The relationship between SDI and incidence rates demonstrated distinct patterns across different regions.

Eastern Europe exhibited the highest incidence rates, reaching approximately 70 per 100,000 population at SDI levels around 0.6-0.7. Central Asia and High-income Asia Pacific regions showed the second-highest peaks, with incidence rates of about 30-40 per 100,000 population at similar SDI levels.

Western Europe and High-income North America displayed moderate incidence rates (20-30 per 100,000 population) at high SDI values (0.8-0.9). Australasia and Southern Latin America followed a similar pattern but with slightly lower rates.

Regions with lower SDI values (0.3-0.5), including South Asia, Southeast Asia, and Sub-Saharan African regions, consistently showed lower incidence rates, generally

below 10 per 100,000 population. The global average curve demonstrated an overall increasing trend with SDI, peaking at moderate SDI levels (0.6-0.7) before showing a slight decline at the highest SDI values.

East Asia and Central Europe showed distinct trajectories, with rates increasing substantially as SDI increased from 0.4 to 0.7, followed by a plateau or slight decrease at higher SDI levels. The relationship between SDI and incidence rates appeared most pronounced in the mid-range of SDI values (0.5-0.7), where the greatest variation in rates was observed across regions.

3.1.2 Regional Association Between SDI and Age-Standardized Mortality Rates

The relationship between socio-demographic index (SDI) and age-standardized death rates (ASR) for acute carbon monoxide poisoning across 21 global regions showed a moderate positive correlation (Spearman's $r = 0.3075$, $p = 7.627e-18$) (Figure 5). The pattern of mortality rates demonstrated distinct regional variations across the SDI spectrum.

Eastern Europe exhibited the highest mortality rates, reaching approximately 7 deaths per 100,000 population at SDI values between 0.6 and 0.7. Central Asia showed the second highest peak, with mortality rates around 3 deaths per 100,000 population at similar SDI levels.

Most regions maintained relatively low mortality rates (below 1 per 100,000 population) across the SDI spectrum. High-income regions, including Western Europe, High-income North America, and Australasia, showed consistently low mortality rates despite their high SDI values (0.8-0.9).

The global average curve demonstrated a subtle increase with rising SDI values up to approximately 0.6, followed by a gradual decline at higher SDI levels. East Asia showed a distinctive pattern with elevated mortality rates at moderate SDI levels (0.6-0.7), while maintaining lower rates than Eastern Europe.

Regions with lower SDI values (0.3-0.5), including South Asia, Southeast Asia, and Sub-Saharan African regions, generally maintained low mortality rates below 0.5 per 100,000 population, showing minimal variation across different SDI values.

3.1.3 Regional Association Between SDI and Age-Standardized DALY Rates

The relationship between socio-demographic index (SDI) and age-standardized DALY rates for acute carbon monoxide poisoning across 21 global regions showed a moderate positive correlation (Spearman's $r = 0.3663$, $p = 3.671e-25$) (Figure 6). The pattern of DALY rates demonstrated marked regional variations across the SDI spectrum.

Eastern Europe exhibited the highest DALY rates, reaching approximately 300 DALYs per 100,000 population at SDI values between 0.6 and 0.7. Central Asia showed the second highest burden, with rates around 150 DALYs per 100,000 population at similar SDI levels.

The global average curve demonstrated a gradual increase with rising SDI values up to approximately 0.6, followed by a decline at higher SDI levels. East Asia showed a distinctive pattern with elevated DALY rates at moderate SDI levels (0.6-0.7), though maintaining lower rates than Eastern Europe.

High-income regions, including Western Europe, High-income North America, and Australasia, showed relatively low DALY rates (below 50 per 100,000 population) despite their high SDI values (0.8-0.9). Most other regions maintained DALY rates below 100 per 100,000 population across the SDI spectrum.

Regions with lower SDI values (0.3-0.5), including South Asia, Southeast Asia, and Sub-Saharan African regions, consistently showed the lowest DALY rates, generally below 25 per 100,000 population, with minimal variation across different SDI values.

3.2 National-Level Analysis

3.2.1 National-Level Analysis of SDI and Age-Standardized Incidence Rates

The relationship between socio-demographic index (SDI) and age-standardized incidence rates (ASR) of acute carbon monoxide poisoning at the national level showed a strong positive correlation (Spearman's $r = 0.7602$, $p < 0.001$) (Figure 7). The analysis across 204 countries revealed distinct patterns of disease burden across the SDI spectrum.

Countries in Eastern Europe demonstrated the highest incidence rates, with the Republic of Moldova, Russian Federation, and Czechia recording rates above 35 per 100,000 population at SDI values between 0.7 and 0.8. These were followed by Belarus, Slovenia, and Estonia, with rates between 30 and 35 per 100,000 population at similar SDI levels.

A second tier of countries, including Latvia, Hungary, Slovakia, and Bosnia and Herzegovina, showed rates between 25 and 30 per 100,000 population, predominantly clustering at SDI values of 0.7-0.8. Nations with very high SDI values (>0.8), such as Canada, Belgium, and Singapore, generally demonstrated moderate incidence rates between 15 and 25 per 100,000 population.

Countries with lower SDI values (<0.5) consistently showed lower incidence rates, typically below 10 per 100,000 population. These included most nations in Sub-Saharan Africa, South Asia, and parts of Southeast Asia. The overall pattern showed a gradual increase in incidence rates with rising SDI values up to

approximately 0.7, after which the relationship became more heterogeneous across countries.

The distribution showed notable regional clustering, with neighboring countries often displaying similar incidence rates despite varying SDI levels, particularly evident in the Eastern European cluster at the higher end of the incidence spectrum and the Sub-Saharan African cluster at the lower end.

3.2.2 National-Level Analysis of SDI and Age-Standardized Mortality Rates

The relationship between socio-demographic index (SDI) and age-standardized death rates (ASR) for acute carbon monoxide poisoning across 204 countries showed a weak positive correlation (Spearman's r = 0.1341, p = 5.584e-02) (Figure 8). The country-level analysis revealed distinct mortality patterns across different SDI levels.

The highest mortality rates were observed in Eastern European nations, with the Republic of Moldova and Russian Federation showing rates above 2 per 100,000 population at SDI values between 0.6 and 0.8. Mongolia and Kazakhstan also demonstrated notably high mortality rates, exceeding 1.5 per 100,000 population at moderate SDI levels.

Countries with high SDI values (>0.8), including Western European nations, North America, and high-income Asian countries, generally maintained low mortality rates below 0.5 per 100,000 population. Notable exceptions included Lithuania and Latvia, which showed relatively higher rates despite their high SDI values.

A cluster of countries with low to moderate SDI values (0.3-0.5) exhibited varying mortality rates. Afghanistan showed a distinctly high rate (approximately 1.8 per 100,000) at a low SDI value, while Nepal and Yemen demonstrated moderate rates (0.8-1.0 per 100,000) in similar SDI ranges.

The majority of countries, particularly those with SDI values below 0.6, maintained mortality rates below 0.5 per 100,000 population. This included most nations in Africa, South Asia, and Southeast Asia, showing minimal variation in mortality rates despite their varying SDI levels.

3.2.3 National-Level Analysis of SDI and Age-Standardized DALY Rates

The relationship between socio-demographic index (SDI) and age-standardized DALY rates for acute carbon monoxide poisoning across 204 countries demonstrated a weak positive correlation (Spearman's r = 0.2233, p = 1.356e-03) (Figure 9). The country-level analysis revealed substantial heterogeneity in DALY burden across SDI levels.

Mongolia showed the highest DALY rate, reaching approximately 120 per 100,000 population at a moderate SDI level. This was followed by the Republic of Moldova and Russian Federation, both recording rates above 100 DALYs per 100,000 population at SDI values between 0.6 and 0.8.

A cluster of countries including Kazakhstan, Ukraine, and Belarus demonstrated DALY rates between 60 and 80 per 100,000 population at moderate to high SDI levels (0.6-0.8). Afghanistan showed notably high DALY rates (approximately 85 per 100,000) despite its low SDI value.

Countries with high SDI values (>0.8), including most Western European nations, North America, and high-income Asian countries, generally maintained low DALY rates below 20 per 100,000 population. Notable exceptions included Lithuania and Latvia, which showed moderately elevated rates despite their high SDI values.

The majority of countries, particularly those with SDI values below 0.6, maintained DALY rates below 40 per 100,000 population. This included most nations in Africa, South Asia, and Southeast Asia, with minimal variation in DALY rates despite their varying SDI levels.

4 Global Trends and Future Projections

4.1 Projected Trends in Age-Standardized Incidence Rates Through 2050

Global age-standardized incidence rate (ASR) of acute carbon monoxide poisoning is projected to continue its historical decline through 2050 (Figure 10). From an observed rate of approximately 12 per 100,000 population in 1990, the ASR decreased to 8 per 100,000 population by 2020. The forecasting model predicts a further reduction to approximately 5 per 100,000 population by 2050, with widening uncertainty intervals in the long-term projection period.

4.2 Projected Trends in Age-Standardized Mortality Rates Through 2050

The global age-standardized mortality rate of acute carbon monoxide poisoning is projected to continue declining through 2050 (Figure 11). The historical trend shows a peak of approximately 0.8 per 100,000 population in the early 1990s, followed by a steady decrease to 0.35 per 100,000 population by 2020. The model forecasts a further reduction to approximately 0.15 per 100,000 population by 2050, with gradually expanding uncertainty intervals in the projection period.

4.3 Projected Trends in Age-Standardized DALY Rates Through 2050

Global age-standardized DALY rates for acute carbon monoxide poisoning are projected to maintain their downward trend through 2050 (Figure 12). From a peak of approximately 40 DALYs per 100,000 population in the early 1990s, the rate declined

to around 15 DALYs per 100,000 population by 2020. The forecasting model predicts a further decrease to approximately 5 DALYs per 100,000 population by 2050, with increasing uncertainty intervals in the latter projection period.

DISCUSSION

This study provides the first comprehensive analysis of the global burden of carbon monoxide (CO) poisoning, encompassing trends in incidence, mortality, and disability-adjusted life years (DALYs) from 1990 to 2021. Our findings reveal an overall declining trend in the global burden of CO poisoning, albeit with significant variations across geographical regions and socio-demographic index (SDI) levels. These results have important implications for developing targeted prevention strategies.

First, our study demonstrates that the global age-standardized incidence rate of CO poisoning decreased from 12.13 per 100,000 in 1990 to 7.87 per 100,000 in 2021, with an average annual decline of 1.16%. This downward trend likely reflects improvements in safety standards during industrialization[15], enhanced indoor ventilation facilities[16], and increased public awareness[17]. However, Eastern Europe and High-income Asia Pacific regions maintain relatively high incidence rates, possibly due to high heating demands during winter months and delayed updates of aging heating infrastructure.

Second, our research reveals a complex relationship between CO poisoning burden and socioeconomic development levels. High-SDI and high-middle SDI regions exhibited the highest incidence rates but relatively lower mortality rates and DALY burdens, suggesting that while these regions report more cases, better medical conditions lead to improved patient outcomes[18, 19]. In contrast, low-SDI regions show lower reported incidence rates but potentially suffer from underreporting and limited medical resources, which may result in poorer outcomes[20, 21]. This aligns with previous findings[6, 8] regarding insufficient acute poisoning treatment capabilities in developing countries.

The geographical distribution analysis reveals significant regional disparities. Eastern Europe consistently maintains the highest burden levels, attributable to its climatic characteristics[22], heating methods, and unique circumstances during socioeconomic transition. The lower reported rates in Africa and Southeast Asia may mask the actual disease burden, indicating a need to strengthen surveillance systems in these regions. Our study is the first to systematically analyze gender disparities, finding that males generally experience higher mortality rates and DALY burdens than females, possibly due to differences in occupational exposure risks and behavioral patterns[23, 24].

Several limitations should be acknowledged in our study. First, the quality of Global Burden of Disease study data varies across countries, with potential incompleteness in low-income nations. Second, our inability to obtain detailed exposure information and

specific causes of death limits concrete guidance for prevention strategies. Third, while our prediction models consider historical trends, they may not fully incorporate potential policy changes and technological advancements.

Looking forward, our forecast analysis indicates that the global burden of CO poisoning will continue to decrease through 2050, though regional disparities may persist. This suggests the need for differentiated prevention strategies: high-burden regions should focus on upgrading heating facilities and enforcing safety standards, while low-income regions need strengthened infrastructure and medical treatment capabilities. Future research should explore the underlying causes of regional differences, evaluate the cost-effectiveness of various prevention measures, and establish more comprehensive surveillance systems. Additionally, the promotion of clean energy and development of smart early warning technologies may further reduce the disease burden of CO poisoning.

The implications of our findings extend beyond public health planning to policy making. The strong correlation between SDI and CO poisoning burden patterns suggests that economic development alone may not guarantee reduced disease burden without targeted interventions. The persistent regional disparities highlight the need for international cooperation in sharing prevention strategies and medical expertise. Moreover, the projected trends through 2050 provide valuable information for long-term healthcare planning and resource allocation.

Our findings also emphasize the importance of considering demographic and socioeconomic factors in prevention strategies. The higher burden among males and in specific geographical regions suggests the need for targeted interventions that consider local contexts and risk factors. The relationship between SDI and disease burden patterns indicates that prevention strategies should be tailored to different development stages, with particular attention to regions undergoing rapid industrialization.

These insights can inform evidence-based policy making and intervention strategies at global, regional, and national levels. Priority should be given to strengthening surveillance systems in low-resource settings, implementing targeted prevention programs in high-burden regions, and developing capacity for acute poisoning treatment in areas with limited healthcare resources. Furthermore, international collaboration should be enhanced to share successful prevention strategies and support regions with limited resources in building their prevention and treatment capabilities.

Funding Information: This research received no specific grant from any funding agency in the public, commercial, or not-for-profit sectors.

Author Contribution: WW conceptualized and designed the study, collected and analyzed the data, interpreted the results, wrote the original draft, and reviewed and edited the final manuscript.

Competing Interests: The author declares no competing interests.

Data Availability: The data that support the findings of this study are available from the Global Health Data Exchange (GHDx) platform (http://ghdx.healthdata.org/). The datasets analyzed during the current study are publicly available.

Ethics Statement: This study was conducted in accordance with the ethical standards of the institutional and/or national research committee and with the 1964 Helsinki Declaration and its later amendments or comparable ethical standards. The need for informed consent was waived by the ethics committee of People’s Hospital of Anji (Approval No. P202309-1)because the study involved the analysis of fully anonymized retrospective data from the Global Burden of Disease Study 2021. All data were accessed after complete anonymization, and no individual identifiers were included in the analysis.

Reference：

1. Dent, M.R., et al., *Carbon Monoxide Poisoning: From Microbes to Therapeutics.* Annual Review of Medicine, 2024. **75**: p. 337-351.
2. Rose, J.J., et al., *Carbon Monoxide Poisoning: Pathogenesis, Management, and Future Directions of Therapy.* American Journal of Respiratory and Critical Care Medicine, 2017. **195**(5): p. 596-606.
3. Hampson, N.B., et al., *Practice Recommendations in the Diagnosis, Management, and Prevention of Carbon Monoxide Poisoning.* American Journal of Respiratory and Critical Care Medicine, 2012. **186**(11): p. 1095-1101.
4. Zazzeron, L., W. Franco, and R. Anderson, *Carbon monoxide poisoning and phototherapy.* Nitric Oxide-Biology and Chemistry, 2024. **146**: p. 31-36.
5. Savioli, G., et al., *Carbon Monoxide Poisoning: From Occupational Health to Emergency Medicine.* Journal of Clinical Medicine, 2024. **13**(9).
6. Long, J.H., et al., *Temporal trends of carbon monoxide poisoning mortality at the global, regional and national levels: a cross-sectional study from the Global Burden of Disease study, 1990 and 2017.* Bmj Open, 2021. **11**(11).
7. Cui, P., et al., *Burden of carbon monoxide poisoning in China, 1990-2019: A systematic analysis of data from the global burden of disease study 2019.* Frontiers in Public Health, 2022. **10**.
8. Moberg, M.E., et al., *Global, regional, and national mortality due to unintentional carbon monoxide poisoning, 2000-2021: results from the Global Burden of Disease Study 2021.* Lancet Public Health, 2023. **8**(11): p. E839-E849.

9. Abbafati, C., et al., *Global burden of 369 diseases and injuries in 204 countries and territories, 1990-2019: a systematic analysis for the Global Burden of Disease Study 2019.* Lancet, 2020. **396**(10258): p. 1204-1222.
10. Stevens, G.A., et al., *Guidelines for Accurate and Transparent Health Estimates Reporting: the GATHER statement.* Lancet, 2016. **388**(10062): p. E19-E23.
11. Anselin, L. and S.J. Rey, *Open Source Software for Spatial Data Science.* Geographical Analysis, 2022. **54**(3): p. 429-438.
12. Kim, H.J., et al., *Permutation tests for joinpoint regression with applications to cancer rates.* Statistics in Medicine, 2000. **19**(3): p. 335-351.
13. Wu, A.M., et al., *Global, regional, and national burden of neck pain, 1990-2020, and projections to 2050: a systematic analysis of the Global Burden of Disease Study 2021.* Lancet Rheumatology, 2024. **6**(3): p. e142-e155.
14. Zhang, S.N., et al., *Current status and trends in thalassemia burden across South, East and Southeast Asia, 1990-2021 a systematic analysis for the global burden of disease study 2021.* Bmc Public Health, 2024. **24**(1).
15. Wang, Y., et al., *Synthesis and Characteristics of a pH-Sensitive Sol-Gel Transition Colloid for Coal Fire Extinguishing.* Gels, 2023. **9**(1).
16. Sami, S., et al., *Investigation of SARS-CoV-2 Transmission Associated With a Large Indoor Convention - New York City, November-December 2021.* MMWR Morb Mortal Wkly Rep, 2022. **71**(7): p. 243-248.
17. Kumar, S., et al., *Pulse-based cropping systems for soil health restoration, resources conservation, and nutritional and environmental security in rainfed agroecosystems.* Front Microbiol, 2022. **13**: p. 1041124.
18. Cerbin, L.P., et al., *Is Time of the Essence? The Impact of Time of Hospital Presentation in Acute Heart Failure: Insights From ASCEND-HF Trial.* JACC Heart Fail, 2018. **6**(4): p. 298-307.
19. Ahn, J., et al., *The Relationship between Hospital Selection by Employer and Disabilities in Occupational Accidents in Korea.* Saf Health Work, 2022. **13**(3): p. 279-285.
20. de Souza, D.K., et al., *A quick and cost effective method for the diagnosis of Mycobacterium ulcerans infection.* BMC Infect Dis, 2012. **12**: p. 8.
21. Li, Y., et al., *Incidence of pulmonary tuberculosis in Chinese adults with type 2 diabetes: a retrospective cohort study in Shanghai.* Sci Rep, 2020. **10**(1): p. 8578.
22. He, B., L. Huang, and Q. Wang, *Precipitation deficits increase high diurnal temperature range extremes.* Sci Rep, 2015. **5**: p. 12004.
23. Huang, Y., et al., *Deciphering genetic causes for sex differences in human health through drug metabolism and transporter genes.* Nat Commun, 2023. **14**(1): p. 175.
24. Oudit, G.Y., et al., *Angiotensin-converting enzyme 2-at the heart of the COVID-19 pandemic.* Cell, 2023. **186**(5): p. 906-922.

Tables:

Table 1: Global Age-Standardized Incidence Rates of Acute Carbon Monoxide Poisoning (1990-2021)

**Characteristics**	**Incidence (95% uncertainty interval)**				
	**Number of cases,1990**	**ASR per 100000 population,1990**	**Number of cases,2021**	**ASR per 100000 population,2021**	**Estimated annual percentage change,1990-2021**
**Location**					
Global	693139.82(469815.01,981111.48)	12.13(8.30,17.00)	294173.15(209197.11,395043.48)	7.87(5.54,10.81)	-1.16(-1.35,-0.96)
**SEX**					
Male	328711.45(224639.10,458237.94)	11.35(7.88,15.56)	294173.15(209197.11,395043.48)	7.60(5.46,10.26)	-1.04(-1.24,-0.84)
Female	364428.36(242796.67,517300.42)	12.92(8.69,18.19)	8306466.88(211120.03,426664.43)	8.16(5.58,11.48)	-1.26(-1.44,-1.07)
**SDI**					
High	198935.21(129323.21,286238.29)	24.77(16.00,35.74)	151650.80(102859.16,211801.98)	17.77(11.48,25.96)	-0.50(-0.81,-0.19)
High middle	256050.68(179270.77,349599.88)	23.58(16.66,31.97)	196799.02(143406.36,255270.12)	17.85(12.82,24.28)	-0.84(-1.08,-0.60)
Middle	154919.60(101287.58,225975.71)	7.86(5.21,11.28)	148982.45(104247.71,206861.25)	6.59(4.60,9.21)	-0.31(-0.52,-0.10)
Low middle	58389.08(34906.93,88761.71)	3.96(2.42,5.90)	63033.75(39297.60,94359.63)	3.08(1.96,4.56)	-0.75(-0.93,-0.57)
Low	23855.82(14849.28,35667.90)	3.39(2.13,4.96)	39546.60(25813.05,57782.10)	2.78(1.87,4.00)	-0.61(-0.73,-0.48)
**GBD region**					
Andean Latin America	17726.94(12142.04,25222.30)	5.20(2.99,8.15)	17856.25(12318.94,24992.04)	5.24(3.14,8.21)	0.23(0.09,0.38)
Australasia	19023.75(10938.11,30400.34)	14.57(9.19,21.82)	18737.08(11288.82,29238.40)	12.53(8.10,18.50)	-0.46(-0.58,-0.35)
Caribbean	45513.59(30788.93,65582.50)	10.07(6.38,15.16)	22885.35(15479.23,32773.32)	9.95(6.27,14.98)	0.10(-0.10,0.30)
Central Asia	146853.21(98903.12,212419.73)	22.89(15.90,32.12)	156104.38(114589.18,204066.93)	18.63(12.78,26.62)	-0.53(-0.70,-0.36)

Central Europe	233.18(135.21,365.42)	39.11(26.39,56.82)	473.67(292.14,713.62)	27.93(17.88,41.60)	-0.88(-1.12,-0.65)
Central Latin America	71937.76(46324.29,103772.32)	13.51(7.64,21.48)	55007.05(37113.48,76629.75)	9.76(5.79,15.24)	-0.89(-1.18,-0.60)
Central sub Saharan Africa	120813.46(88810.99,155630.71)	2.72(1.77,3.90)	67288.99(51320.35,84904.71)	2.31(1.61,3.29)	-0.54(-0.61,-0.47)
East Asia	2812.58(1789.11,4158.10)	11.17(7.70,15.68)	3239.31(2150.70,4572.90)	12.87(9.26,17.06)	0.97(0.71,1.24)
Eastern Europe	50421.41(33402.82,72531.56)	55.71(41.19,72.97)	22839.71(15164.33,32314.86)	37.98(28.51,49.23)	-1.65(-2.08,-1.22)
Eastern sub Saharan Africa	12807.52(7926.84,19414.28)	3.59(2.36,5.24)	15865.56(10592.01,22946.20)	2.76(1.92,3.87)	-0.88(-0.98,-0.77)
High-income Asia Pacifc	65618.56(40399.00,96858.55)	32.77(21.77,46.85)	64048.97(42794.83,90451.93)	19.63(12.42,29.50)	-1.61(-1.75,-1.47)
High-income North America	3981.36(2506.79,6053.51)	24.71(15.13,37.26)	4327.88(2744.06,6476.59)	20.23(13.00,29.09)	0.64(-0.16,1.44)
North Africa and Middle East	2487.97(1394.06,3978.93)	6.82(4.46,9.79)	3473.55(2091.08,5451.20)	6.27(4.20,8.89)	-0.28(-0.45,-0.11)
Oceania	9773.97(3624.09,18548.81)	2.90(1.72,4.42)	5684.71(3070.07,9373.61)	2.98(1.85,4.43)	0.24(0.05,0.44)
South Asia	28028.39(15540.38,45387.22)	2.86(1.66,4.36)	23333.69(13951.02,36344.19)	1.98(1.18,3.01)	-1.16(-1.40,-0.92)
Southeast Asia	2092.18(1338.52,3038.87)	3.39(2.00,5.35)	3851.93(2531.49,5752.41)	2.84(1.71,4.45)	-0.41(-0.67,-0.15)
Southern Latin America	29249.23(18928.36,42828.76)	24.92(15.47,37.50)	40452.74(26969.25,57497.74)	26.73(17.66,38.98)	0.46(0.19,0.72)
Southern Sub-Saharan Africa	40203.90(22969.17,62557.45)	4.61(3.09,6.60)	37456.27(21965.29,57577.96)	3.04(2.13,4.28)	-1.41(-1.48,-1.34)
Tropical Latin America	9474.21(6130.21,14074.91)	5.60(2.21,10.38)	13963.65(9202.91,20591.85)	2.71(1.42,4.52)	-2.05(-2.24,-1.86)
Western Europe	10891.55(6953.62,16251.54)	22.04(13.87,32.09)	21212.82(13837.67,30711.96)	17.08(11.03,24.98)	-0.60(-0.73,-0.46)
Western Sub-Saharan Africa	3195.10(2105.60,4624.59)	3.89(2.49,5.73)	2536.50(1757.36,3581.84)	3.15(2.09,4.52)	-0.64(-0.83,-0.46)

Table 2: Global Age-Standardized Mortality Rates of Acute Carbon Monoxide Poisoning (1990-2021)

**Characteristics**	**Deaths (95% uncertainty interval)**				
	**Number of cases,1990**	**ASR per 100000 population,1990**	**Number of cases,2021**	**ASR per 100000 population,2021**	**Estimated annual percentage change,1990-2021**
**Location**					
Global	36816.05(31520.91,44285.07)	0.76(0.66,0.91)	28946.78(19894.89,33510.18)	0.35(0.24,0.40)	-2.79(-3.14,-2.44)
**SEX**					
Male	25574.21(20701.72,32318.68)	1.07(0.88,1.39)	20168.71(14337.62,25421.30)	0.50(0.36,0.63)	-2.756(-3.12,-2.39)
Female	11241.85(7609.71,15551.99)	0.46(0.32,0.63)	8778.07(4290.75,10787.13)	0.20(0.10,0.25)	-2.771(-3.09,-2.46)
**SDI**					
High	1054.73(439.33,1779.22)	0.42(0.39,0.44)	1165.51(580.18,1533.12)	0.22(0.20,0.23)	-1.85(-2.07,-1.62)
High middle	508.27(489.00,528.85)	1.79(1.66,2.05)	395.61(358.88,441.29)	0.76(0.59,0.86)	-3.37(-3.93,-2.80)
Middle	794.06(507.64,963.83)	0.73(0.54,1.03)	313.24(282.27,404.35)	0.40(0.21,0.49)	-1.75(-2.00,-1.50)
Low middle	1274.36(1162.78,1361.64)	0.20(0.11,0.27)	927.56(818.72,1070.26)	0.12(0.07,0.16)	-1.89(-2.03,-1.74)
Low	136.15(40.82,193.44)	0.29(0.16,0.45)	140.26(68.54,238.92)	0.18(0.12,0.32)	-1.62(-1.71,-1.54)
**GBD region**					
Andean Latin America	11.13(5.36,16.71)	0.03(0.02,0.05)	45.58(30.48,57.32)	0.07(0.05,0.09)	2.42(1.83,3.02)

Australasia	23.82(22.60,24.97)	0.11(0.11,0.12)	28.17(26.67,29.72)	0.08(0.08,0.09)	-1.70(-2.48,-0.92)
Caribbean	73.63(53.89,104.69)	0.22(0.16,0.29)	44.33(29.46,59.88)	0.09(0.06,0.13)	-2.52(-2.96,-2.08)
Central Asia	1274.36(1162.78,1361.64)	2.06(1.90,2.19)	927.56(818.72,1070.26)	0.98(0.86,1.12)	-3.79(-4.50,-3.07)
Central Europe	2041.25(1912.23,2153.07)	1.58(1.48,1.67)	583.63(538.76,631.37)	0.35(0.33,0.38)	-5.15(-5.42,-4.89)
Central Latin America	508.27(489.00,528.85)	0.34(0.33,0.35)	395.61(358.88,441.29)	0.16(0.14,0.17)	-2.61(-2.82,-2.39)
Central sub Saharan Africa	68.41(34.12,166.40)	0.18(0.09,0.44)	77.66(15.49,386.59)	0.09(0.02,0.45)	-2.12(-2.47,-1.76)
East Asia	14111.63(10243.22,21401.62)	1.30(0.94,1.97)	13588.38(6818.19,17624.96)	0.79(0.40,1.01)	-1.36(-1.68,-1.04)
Eastern Europe	10975.80(10709.96,11176.15)	4.44(4.33,4.52)	5642.24(5227.16,6079.50)	2.10(1.95,2.25)	-3.42(-4.31,-2.53)
Eastern sub Saharan Africa	268.17(140.25,690.06)	0.21(0.10,0.52)	208.84(54.06,978.01)	0.08(0.02,0.37)	-3.06(-3.36,-2.76)
High-income Asia Pacifc	794.06(507.64,963.83)	0.44(0.27,0.53)	313.24(282.27,404.35)	0.12(0.11,0.16)	-3.49(-3.97,-3.02)
High-income North America	822.71(800.43,837.81)	0.27(0.26,0.28)	1303.77(1255.79,1345.75)	0.31(0.30,0.32)	1.16(0.88,1.45)
North Africa and Middle East	2471.39(1433.07,3282.09)	0.82(0.50,1.07)	2770.75(1413.49,3626.76)	0.48(0.25,0.62)	-1.63(-1.73,-1.53)
Oceania	14.76(5.98,24.62)	0.26(0.11,0.46)	24.81(10.87,44.74)	0.20(0.09,0.36)	-0.98(-1.03,-0.94)
South Asia	1054.73(439.33,1779.22)	0.11(0.05,0.17)	1165.51(580.18,1533.12)	0.07(0.03,0.09)	-1.49(-1.59,-1.38)
Southeast Asia	578.41(181.28,821.89)	0.15(0.04,0.21)	712.22(275.08,974.81)	0.10(0.04,0.14)	-1.25(-1.30,-1.21)
Southern Latin America	121.45(103.39,131.14)	0.25(0.21,0.27)	245.89(232.87,258.93)	0.33(0.31,0.35)	0.77(0.04,1.50)
Southern Sub-Saharan Africa	136.15(40.82,193.44)	0.25(0.08,0.36)	140.26(68.54,238.92)	0.17(0.08,0.29)	-1.37(-1.52,-1.22)
Tropical Latin America	81.19(77.22,85.26)	0.06(0.06,0.06)	68.41(65.13,71.53)	0.03(0.03,0.03)	-2.24(-2.66,-1.82)
Western Europe	1262.70(1210.56,1295.79)	0.28(0.27,0.29)	536.83(500.25,560.65)	0.09(0.08,0.09)	-3.59(-3.94,-3.24)
Western Sub-Saharan Africa	122.05(52.76,204.83)	0.12(0.05,0.19)	123.09(73.44,368.12)	0.05(0.03,0.14)	-2.93(-3.22,-2.65)

Table 3: Global Age-Standardized DALY Rates of Acute Carbon Monoxide Poisoning (1990-2021)

**Characteristics**	**DALYs (95% uncertainty interval)**				
	**Number of cases,1990**	**ASR per 100000 population,1990**	**Number of cases,2021**	**ASR per 100000 population,2021**	**Estimated annual percentage change,1990-2021**
**Location**					
Global	1991539.89(1664186.66,2374749.90)	37.59(31.75,44.76)	1228861.20(867811.13,1414898.57)	15.22(10.67,17.57)	-3.18(-3.51,-2.84)
SEX					
Male	1371568.09(1084724.59,1717161.87)	51.84(41.59,64.96)	869791.56(622356.12,1085567.94)	21.52(15.32,26.87)	-3.13(-3.49,-2.77)
Female	619971.79(404516.50,837833.47)	23.13(15.24,31.26)	359069.63(185060.10,427811.11)	8.94(4.58,10.63)	-3.23(-3.53,-2.93)
**SDI**					
High	204572.05(189656.73,216175.29)	22.79(21.03,24.10)	131482.24(121542.74,141061.18)	11.40(10.56,12.19)	-1.89(-2.12,-1.67)
High middle	917444.00(842240.63,1063693.98)	87.55(80.24,100.93)	479786.22(383939.91,542794.21)	33.83(26.92,38.06)	-3.62(-4.17,-3.06)
Middle	670765.64(495400.21,912876.80)	37.82(27.78,51.51)	414933.91(228494.85,511805.42)	16.70(9.17,20.34)	-2.53(-2.77,-2.29)
Low middle	130396.47(63231.17,193448.73)	10.69(5.41,15.27)	120411.32(67754.07,164817.72)	6.23(3.53,8.53)	-2.02(-2.15,-1.89)
Low	66398.32(34227.76,110234.62)	13.07(7.31,20.32)	81550.42(53040.20,132928.44)	7.89(5.21,13.35)	-1.69(-1.74,-1.63)
**GBD region**					
Andean Latin America	820630.90(598757.83,1217182.22)	2.17(1.32,3.02)	511718.87(265448.76,653682.47)	3.83(2.67,4.80)	1.91(1.35,2.48)
Australasia	5377.19(5078.92,5788.58)	7.30(6.85,7.86)	3630.56(3436.15,3861.47)	5.05(4.71,5.46)	-1.80(-2.49,-1.11)

Caribbean	44326.87(42618.63,46385.80)	13.24(9.70,19.61)	60909.71(58475.04,63353.25)	6.40(4.17,8.82)	-2.12(-2.53,-1.70)
Central Asia	96370.67(90201.29,102554.55)	104.85(95.85,112.26)	21108.45(19478.90,22867.45)	46.49(41.04,54.26)	-3.97(-4.66,-3.28)
Central Europe	70670.56(30531.07,124491.55)	78.78(73.81,84.02)	66611.15(36220.66,88601.96)	16.08(14.79,17.47)	-5.40(-5.67,-5.14)
Central Latin America	34257.43(32624.17,36216.62)	19.28(18.46,20.23)	20849.21(19029.68,23084.47)	8.14(7.40,9.05)	-2.76(-2.95,-2.57)
Central sub Saharan Africa	33379.17(11953.46,47448.27)	7.63(4.13,18.03)	37231.89(15737.43,51304.60)	3.83(0.98,17.87)	-2.29(-2.60,-1.97)
East Asia	46356.65(31026.47,55143.99)	67.98(49.48,100.37)	14237.66(12593.92,17902.02)	34.61(17.91,43.44)	-2.10(-2.42,-1.78)
Eastern Europe	71323.25(64693.73,77010.81)	212.93(207.51,217.50)	45707.49(40263.33,53471.48)	95.02(88.66,101.06)	-3.56(-4.44,-2.67)
Eastern sub Saharan Africa	158136.89(88173.65,218144.28)	8.65(4.65,21.42)	147819.73(77885.88,199152.04)	3.40(1.13,14.75)	-3.11(-3.42,-2.80)
High-income Asia Pacifc	497098.53(484659.98,507418.72)	26.55(17.34,31.73)	222147.26(206162.01,237843.44)	7.15(6.30,9.15)	-3.72(-4.14,-3.29)
High-income North America	922.24(359.49,1595.04)	15.23(14.65,15.93)	1547.51(698.44,2694.01)	16.20(15.58,16.84)	1.02(0.70,1.33)
North Africa and Middle East	4872.01(3490.01,7457.12)	43.61(25.48,57.75)	2887.44(1938.23,3912.98)	23.53(12.42,31.64)	-1.92(-2.04,-1.80)
Oceania	16798.08(9169.44,38581.06)	13.41(5.53,22.21)	11857.83(4001.58,51031.32)	10.56(4.68,18.57)	-0.77(-0.82,-0.72)
South Asia	6033.15(3261.37,9495.43)	6.04(2.71,10.04)	7194.91(4794.19,17292.55)	3.54(1.90,4.70)	-1.66(-1.81,-1.50)
Southeast Asia	7581.29(6452.38,8359.07)	7.37(2.62,10.42)	11986.36(11283.92,12755.88)	5.08(2.18,6.98)	-1.22(-1.27,-1.17)
Southern Latin America	4310.08(1893.94,10214.44)	15.18(12.93,16.74)	4247.92(1122.04,18970.74)	17.91(16.80,19.09)	0.42(-0.26,1.10)
Southern Sub-Saharan Africa	1520.86(1426.32,1636.44)	16.45(5.55,23.04)	1547.84(1438.41,1673.98)	11.24(5.62,18.63)	-1.34(-1.53,-1.14)
Tropical Latin America	9749.06(3217.44,13696.04)	3.51(3.32,3.76)	9405.91(4677.61,15655.28)	1.54(1.45,1.64)	-2.44(-2.80,-2.07)
Western Europe	60940.55(57943.42,64260.40)	15.45(14.70,16.26)	23645.84(21516.11,26366.24)	4.99(4.53,5.56)	-3.36(-3.67,-3.05)
Western Sub-Saharan Africa	884.46(521.45,1289.67)	4.60(2.36,7.34)	2567.65(1796.32,3211.73)	2.11(1.38,5.41)	-2.59(-2.79,-2.38)

Figures:

Figure 1: Geographic Distribution of Age-Standardized Incidence Rates of Acute Carbon Monoxide Poisoning

Figure 2: Geographic Distribution of Age-Standardized Mortality Rates of Acute Carbon Monoxide Poisoning

Figure 3: Geographic Distribution of Age-Standardized DALY Rates of Acute Carbon Monoxide Poisoning

Figure 4: Association Between Socio-demographic Index and Age-Standardized Incidence Rates Across 21 Global Regions

Figure 5: Association Between Socio-demographic Index and Age-Standardized Mortality Rates Across 21 Global Regions

Figure 6: Association Between Socio-demographic Index and Age-Standardized DALY Rates Across 21 Global Regions

Figure 7: Association Between Socio-demographic Index and Age-Standardized Incidence Rates Across 204 Countries

Figure 8: Association Between Socio-demographic Index and Age-Standardized Mortality Rates Across 204 Countries

Figure 9: Association Between Socio-demographic Index and Age-Standardized DALY Rates Across 204 Countries

Figure 10: Projected Trends in Age-Standardized Incidence Rates of Acute Carbon Monoxide Poisoning Through 2050

Figure 11: Projected Trends in Age-Standardized Mortality Rates of Acute Carbon Monoxide Poisoning Through 2050

Figure 12: Projected Trends in Age-Standardized DALY Rates of Acute Carbon Monoxide Poisoning Through 2050

Click here to access/download;Figure;Figure 1 .pdf

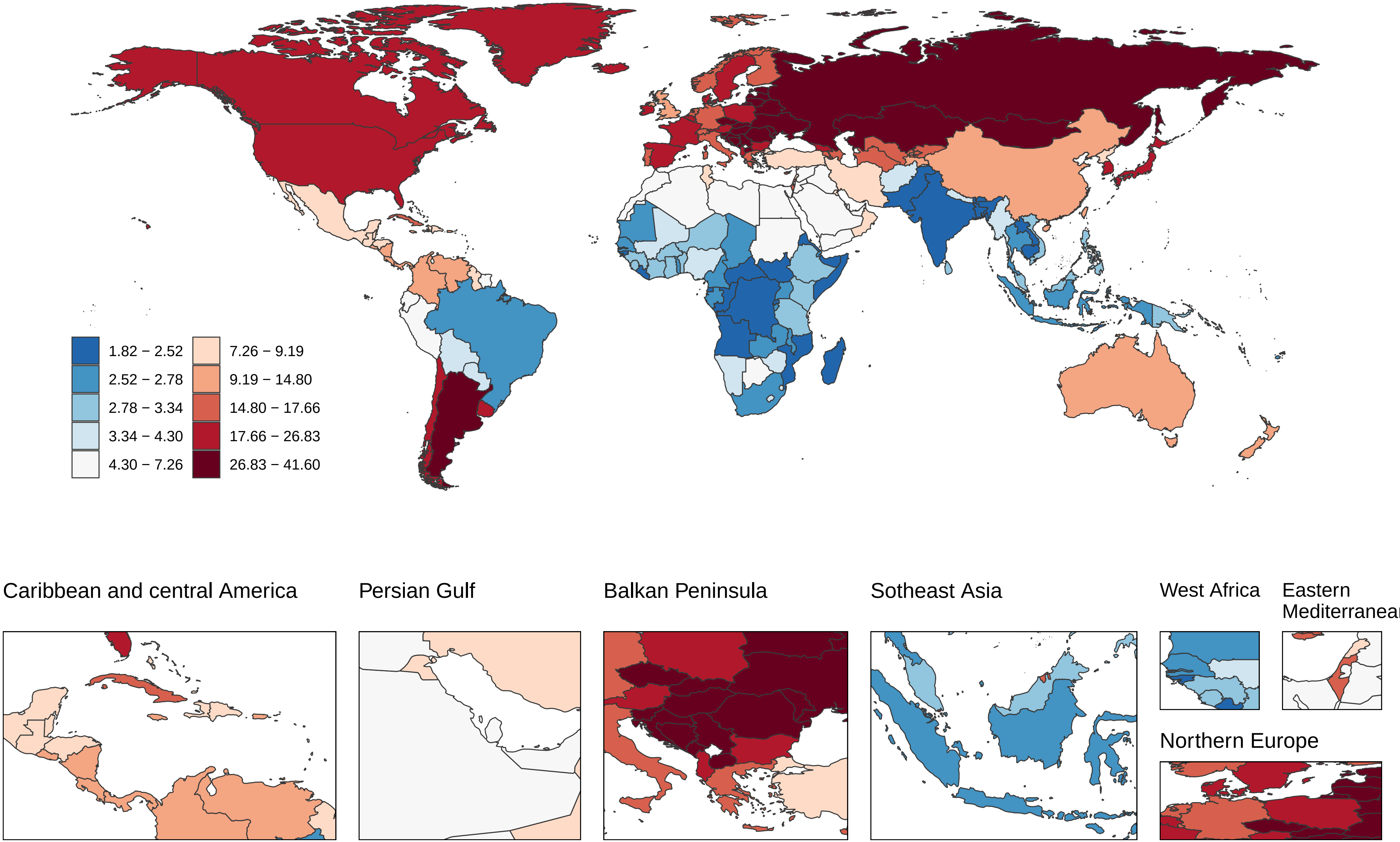

Click here to access/download;Figure;Figure 2 p.pdf

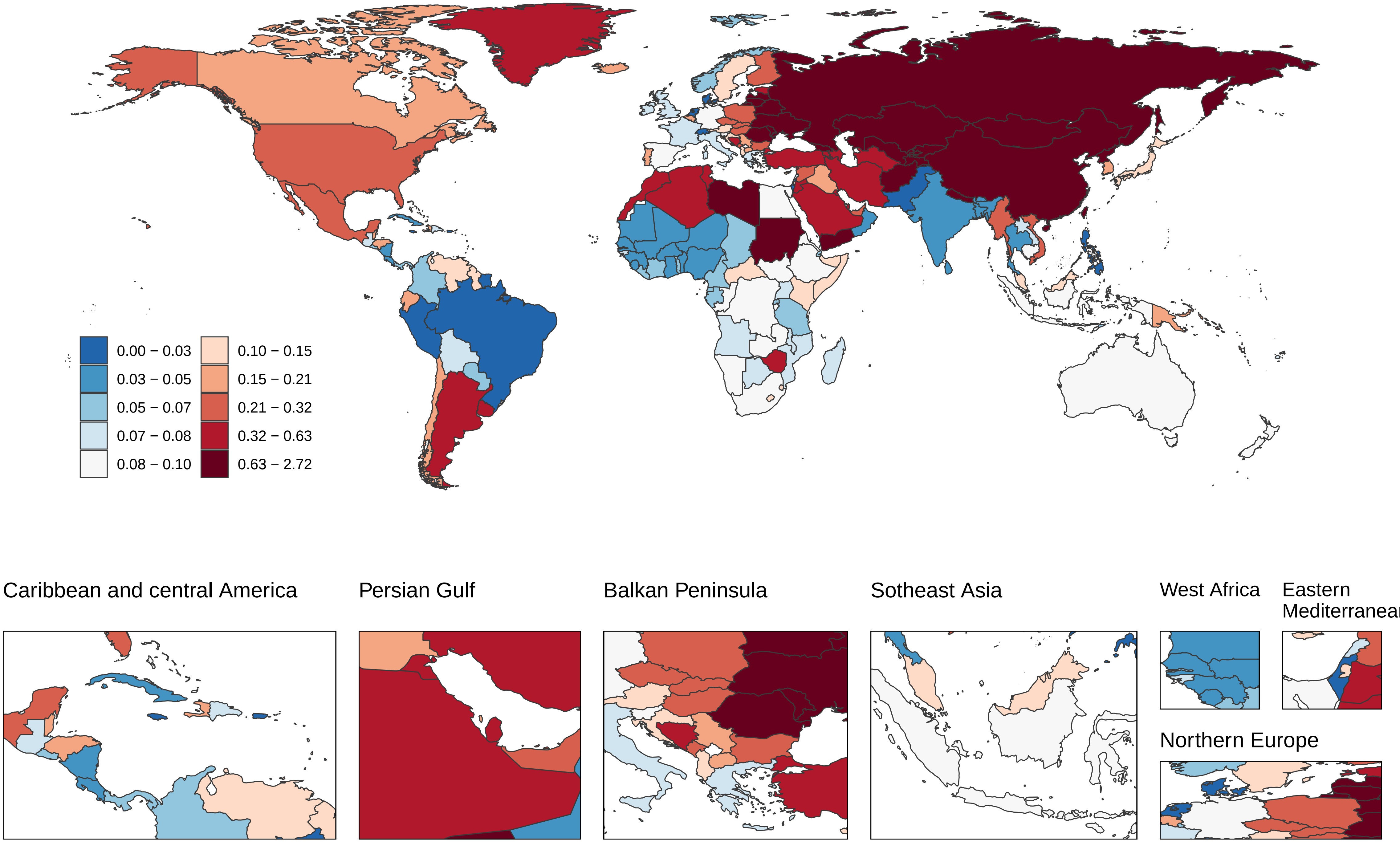

Click here to access/download;Figure;Figure 3.pdf

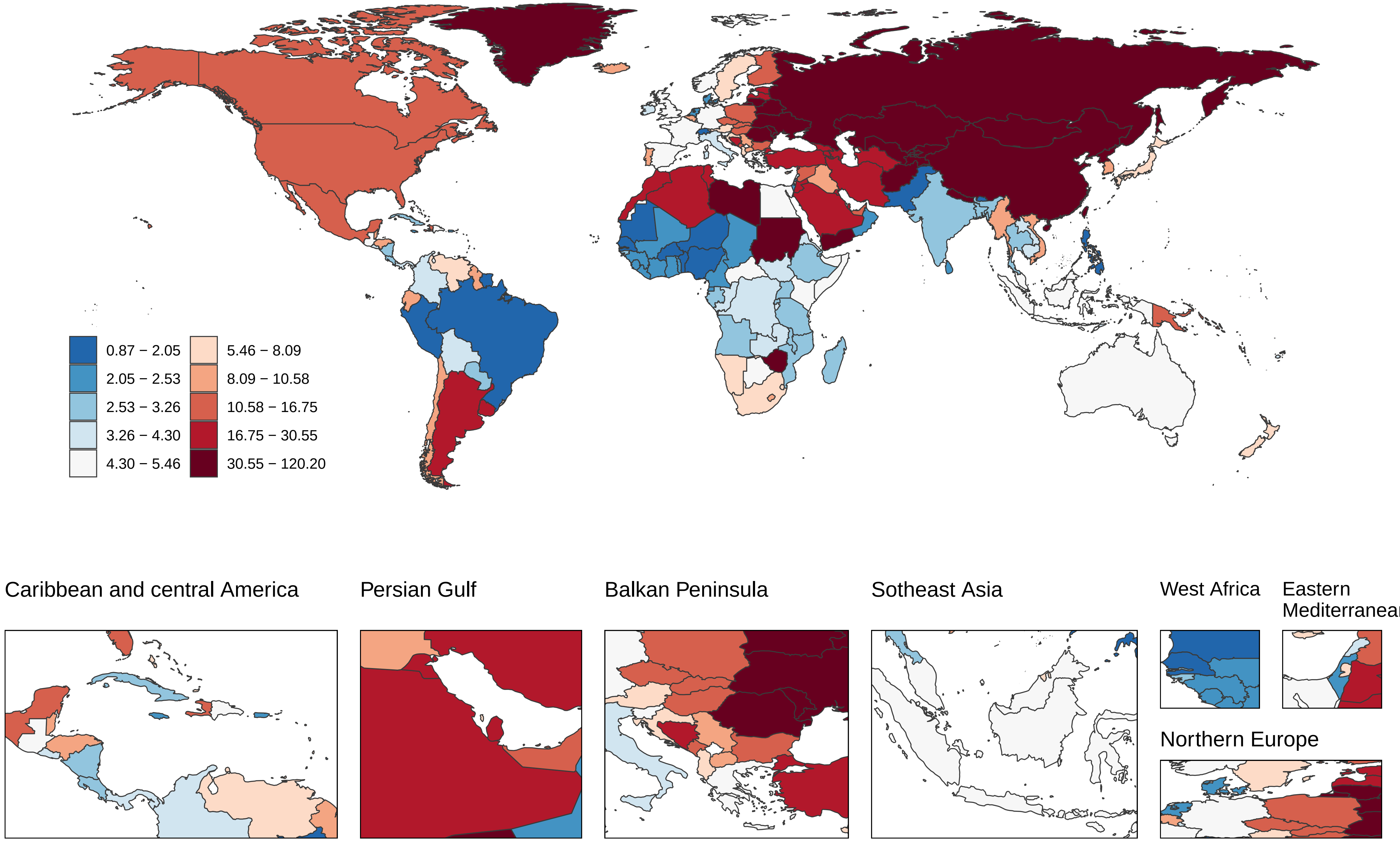

Click here to access/download;Figure;Figure 4.pdf

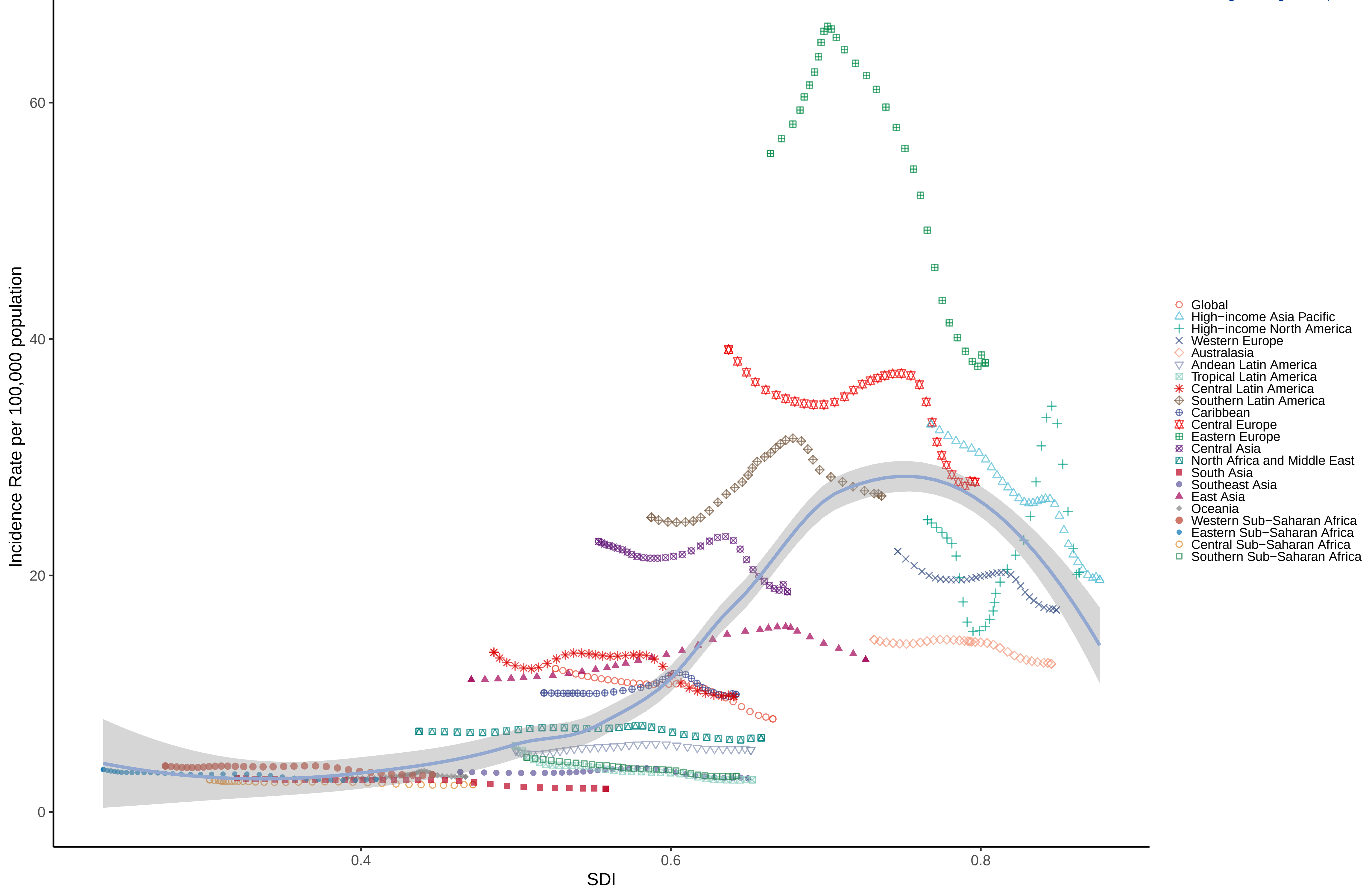

Click here to access/download;Figure;Figure 5.pdf

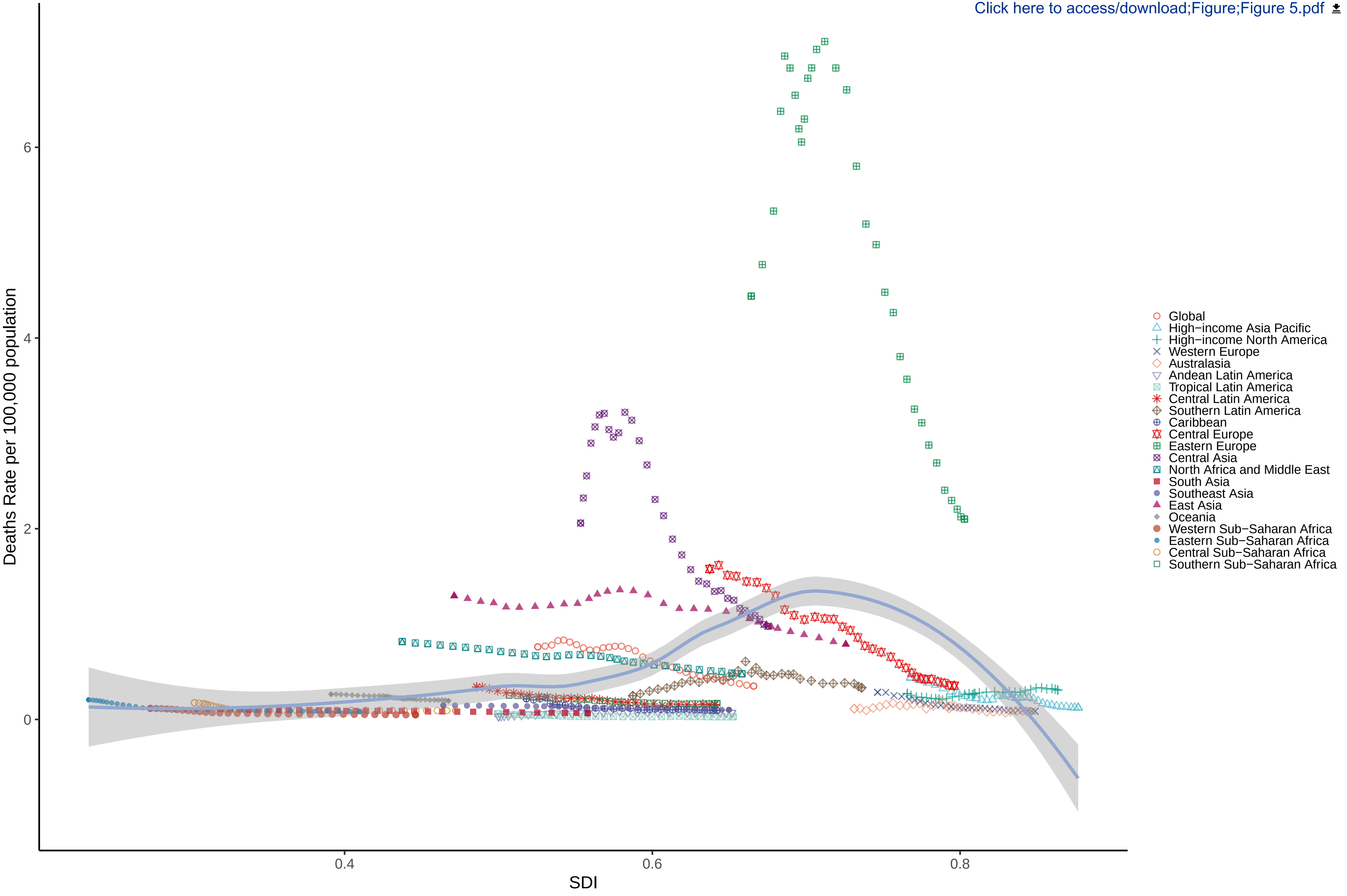

Click here to access/download;Figure;Figure 6.pdf

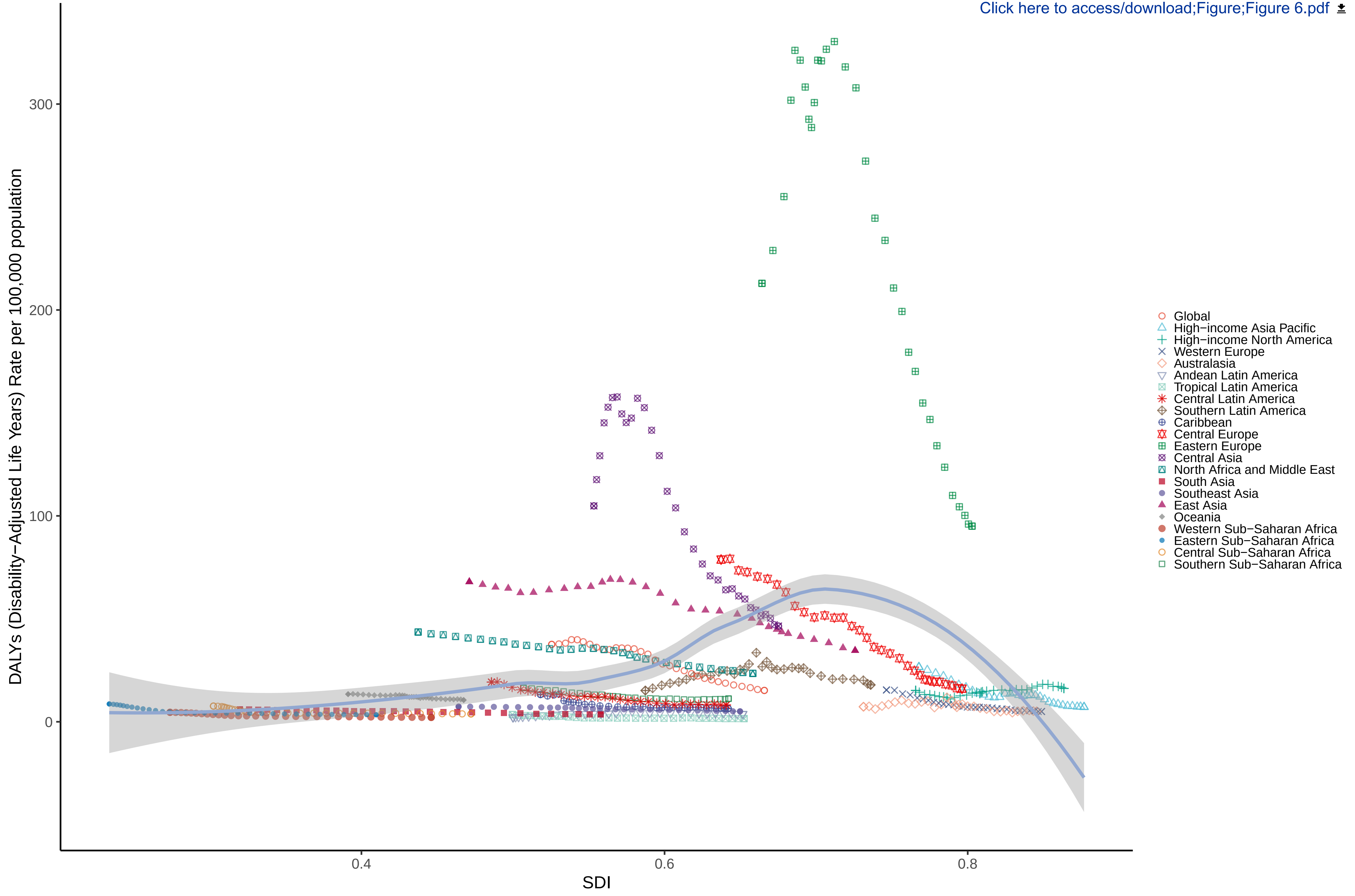

Click here to access/download;Figure;Figure 7.pdf

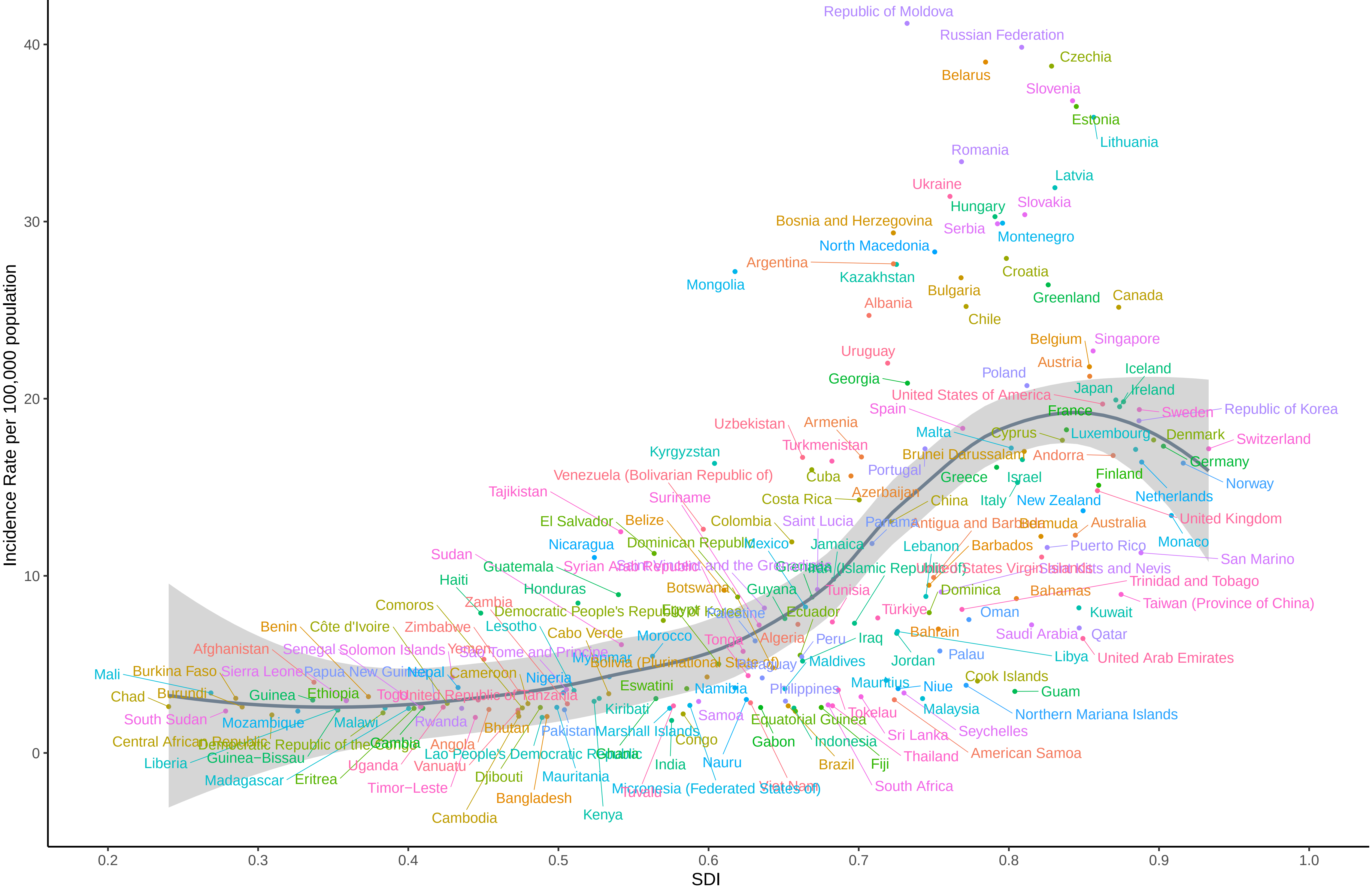

Click here to access/download;Figure;Figure 8.pdf

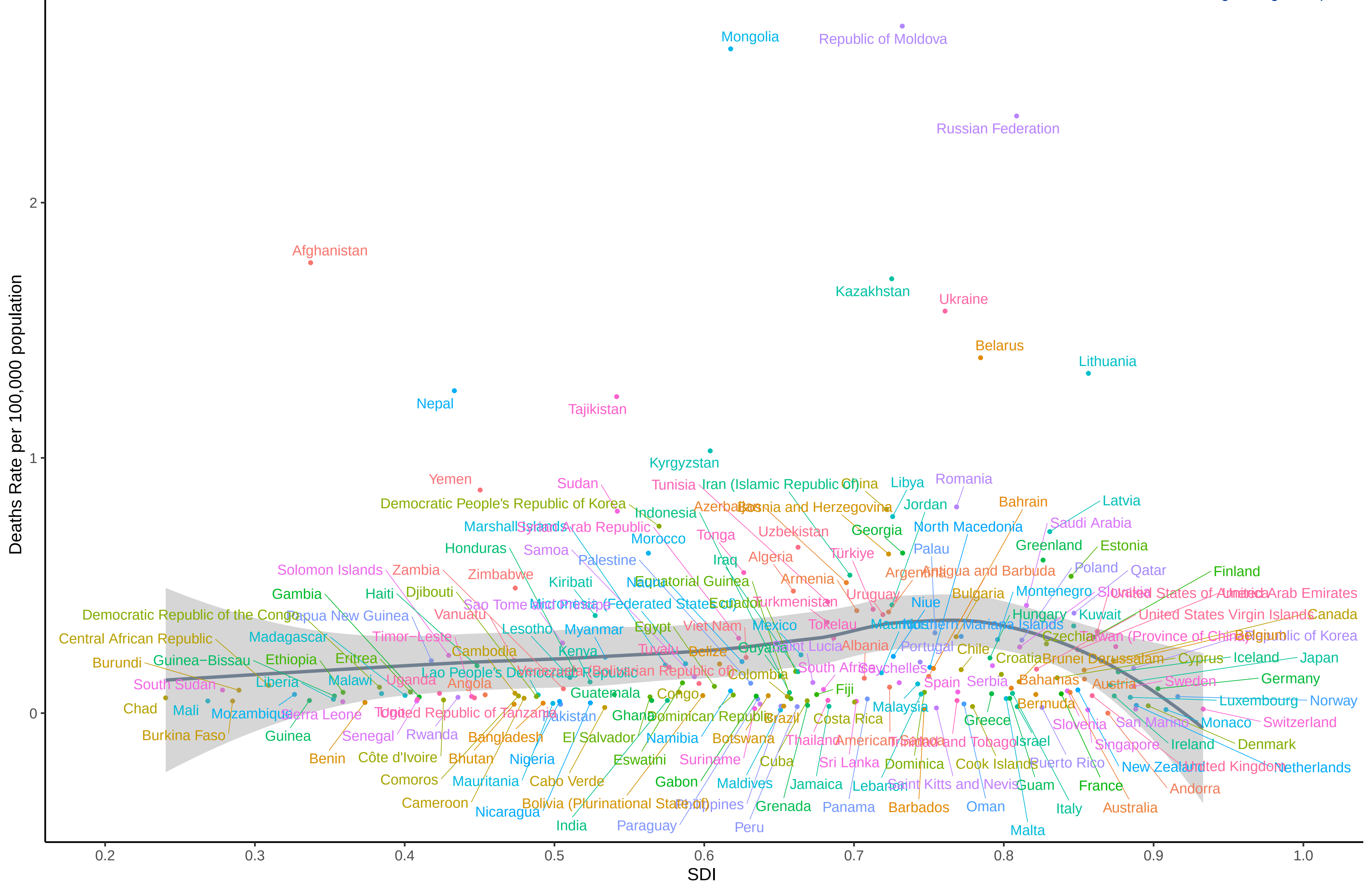

Click here to access/download;Figure;Figure 9.pdf

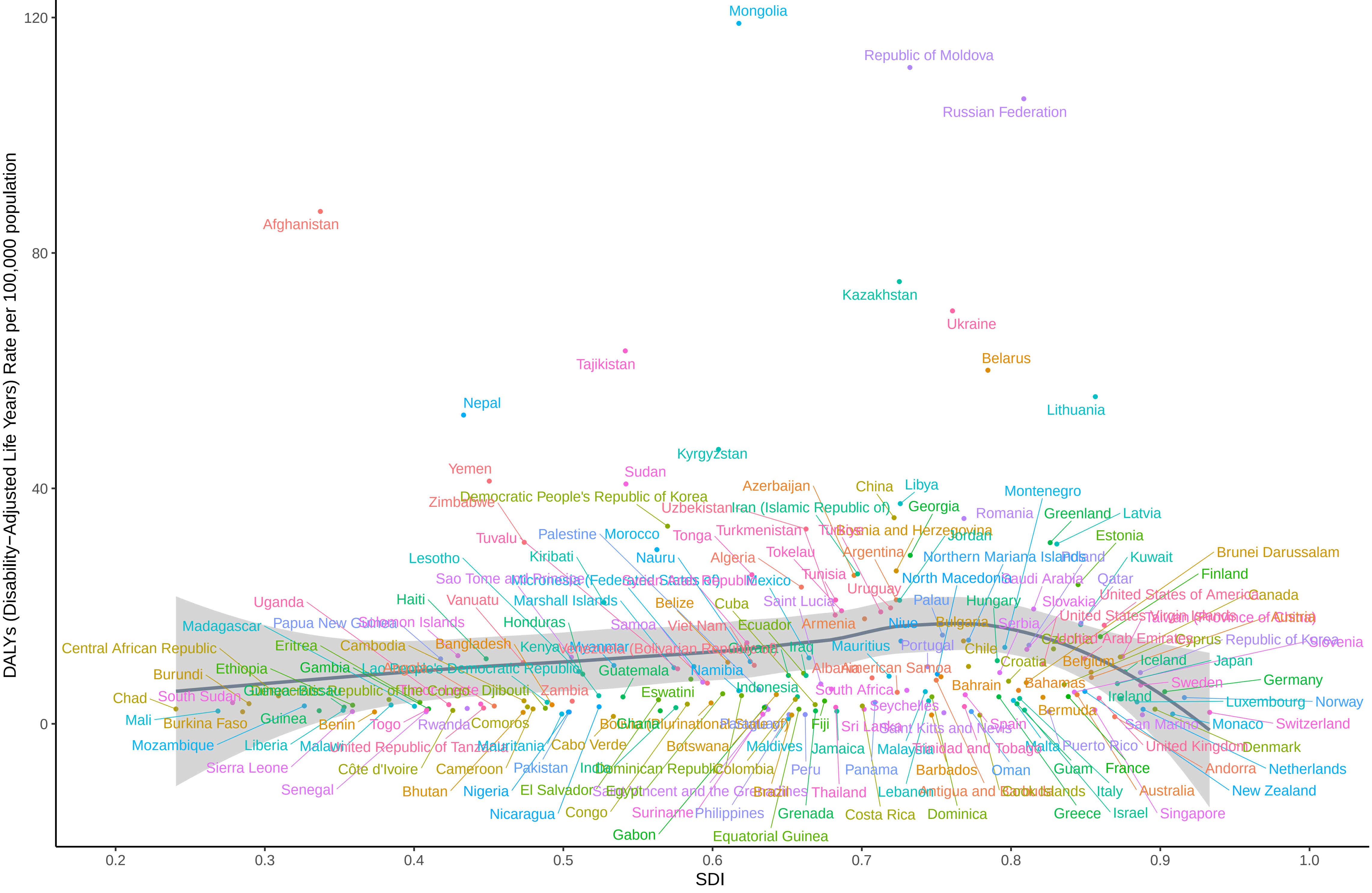

Click here to access/download;Figure;Figure 10.pdf

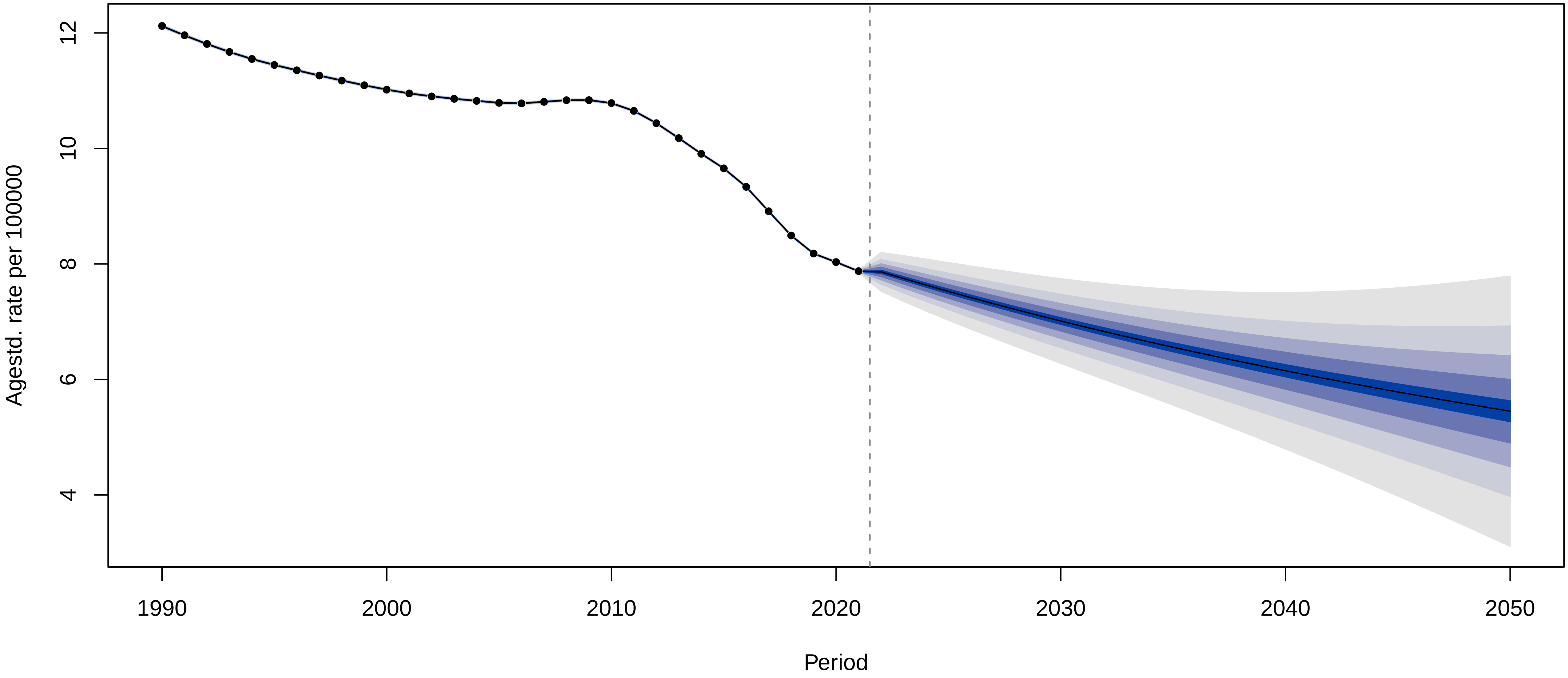

Click here to access/download;Figure;Figure 11.pdf

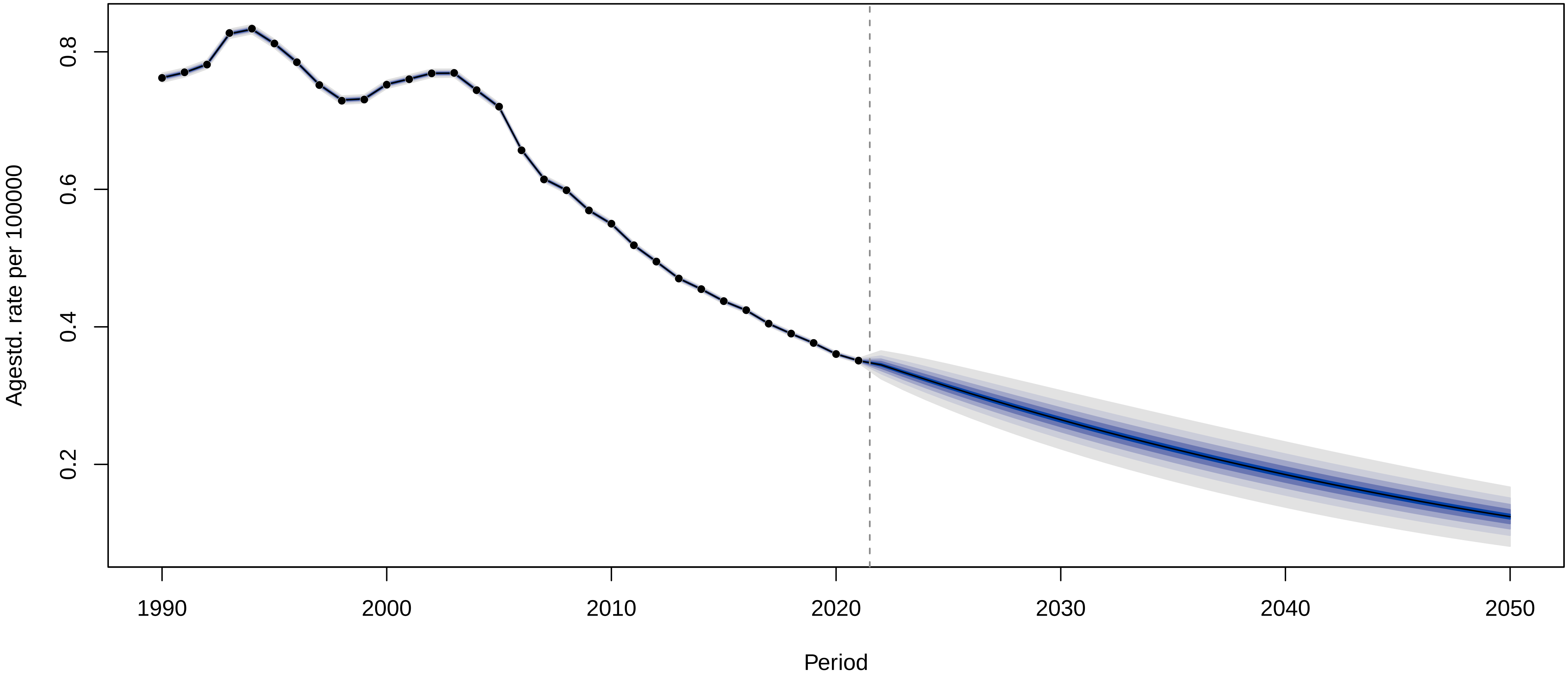

Click here to access/download;Figure;Figure 12.pdf

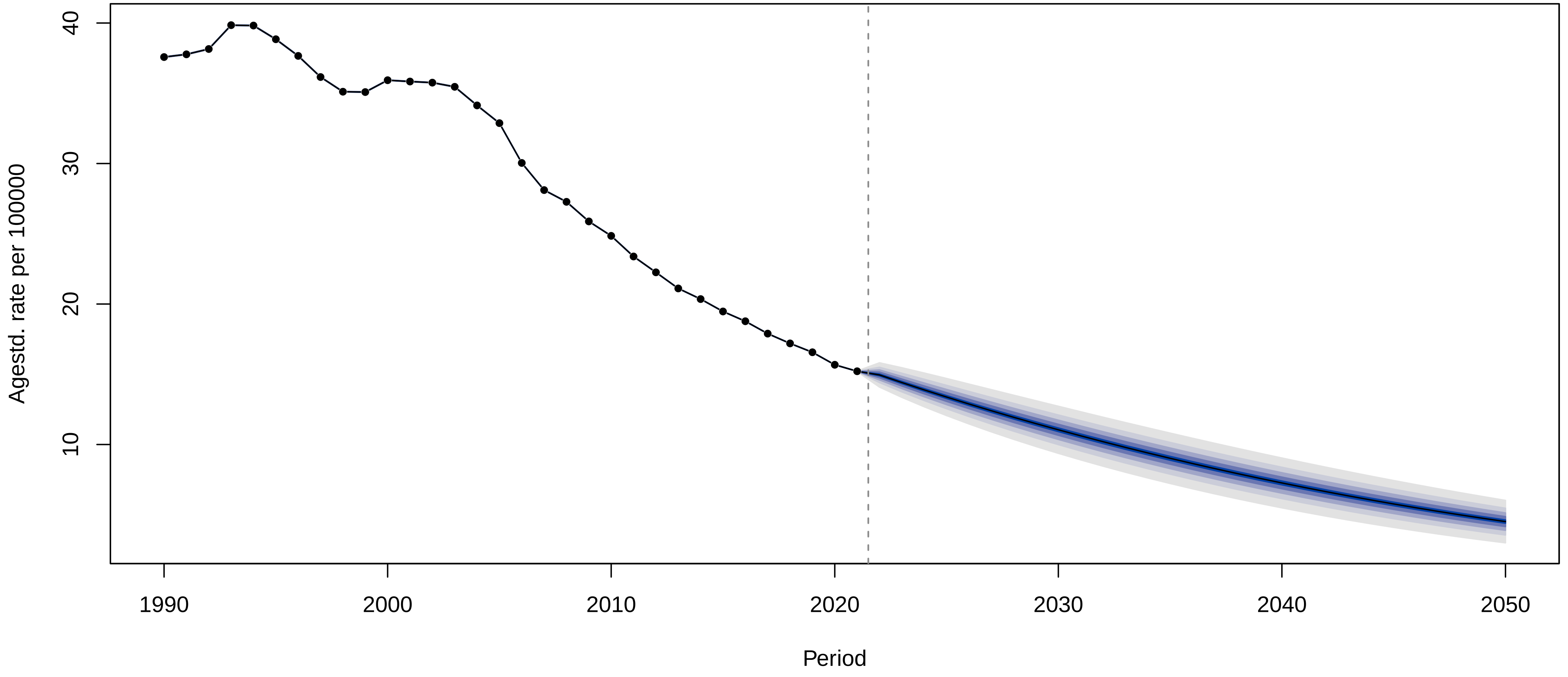

安吉县人民医院医学伦理委员会科学研究审查批件

伦理第（P20230902-1）号

项目名称	早期生活及医疗干预对降低生活性一氧化碳中毒发病率及严重程度的临床研究		
项目来源	湖州市科技局		
项目负责人	王卫广	申请科室	急诊科
审查类别	科研项目	审查方式	会议审查
审查日期	20230902	审查地点	安吉县人民医院
审查文件清单	1. 初始审查申请表（版本号：001 版本日期：20230902）<br>2. 研究方案（版本号：001 版本日期：20230902）<br>3. 知情同意书（版本号：001 版本日期：20230902）<br>4. 研究者简历		
审查人员	戴美琴、王小正、陈秀堂、黄健飞、凌谷燕、曹文娟、王丽娜、郭云潮、陈宏彪		
审评意见	经本伦理委员会审查，**批准**实施该科学研究。<br>该科学研究将接受伦理委员会的跟踪审查？ ☑ 是 ☐ 否<br>审查频率为批准之日起：☐ 3月 ☐ 6个月 ☑ 12个月<br>伦理委员会有根据实际进展情况改变跟踪审查频率的权利。<br>主任委员签名：王拉 日期：2023.9.2		

注意：（请仔细阅读）

请遵循国内外相关法律法规《医疗卫生机构开展临床研究项目管理办法》、《涉及人的生物医学研究伦理审查办法》、WMA（赫尔辛基宣言）和 CIOMS《人体生物医学研究国际道德指南》等的伦理原则。

1. 本批件有效期为一年，无论研究开始与否，请在跟踪审查日到期前 1 个月提交跟踪审查材料，须得到伦理委员会审查同意延长批件有效期后方可继续进行。

2. 请遵循经本伦理委员会批准的方案开展该科学研究，对研究方案，知情同意书和招募材料等的任何修改及项目负责人更换等，须得到伦理委员会审查同意后方可实施。

3. 在本院发生的严重不良事件及影响研究风险受益比的非预期事件请及时报告本伦理委员会。

4. 发现违背试验方案情况须及时报告本伦理委员会。

5. 暂停或提前终止该研究，应及时提交暂停或终止报告。完成研究，请提交研究完成报告供本伦理委员会审查。

地址：安吉县天目中路 699 号 邮编：313300 电话：5034555 转 8552 联系人：曹文娟 邮箱：ajxrmyyll@163.com

	部门：安吉县人民医院医学伦理委员会	文件编号：AF-SC-08(1.0)	
	文件名称：伦理审查委员会伦理审查批件	版本号：1.0	页码：1/2

安吉县人民医院医学伦理委员会伦理审查批件

Ethics Committee Approval Letter of Biomedical Research Involving Humans

批件号 Approval NO.：P20230902-1-2

项目名称 Study Title	早期生活及医疗干预对降低生活性一氧化碳中毒发病率及严重程度的临床研究		
申办方/资助方 Sponsor/Funder	湖州市科技局		
受理号 Acceptance Number	P20230902-1		
主要研究者 Principal Investigator	王卫广	承担科室 Responsible Department	急诊科
审查类别 Category of Review	科研项目	审查方式 Type of Review	快速审查
审查日期 Date of Review	20240801	审查地点 Location of Review	伦理委员会办公室
审查文件清单 Items Reviewed	研究进展报告		
审评意见 Evaluation	同意		
审查决定 Decision	委员会对该项目的审查决定为： ☑批准 (Approval)		
主任/副主任委员签字 Chair Signature	[illegible]		
签发日期 Date of issue	2024. 8.		
伦理审查委员会 Stamp of EC	伦理审查委员会盖章		
联系方式 contact information	电话：0572-5665621 邮箱：ajxrmyyyxllwyh@163.com<br>地址：浙江省湖州市安吉县昌硕街道天目中路 699 号		
批件有效期 Period of Validity	自本伦理审查委员会初始审查批准之日起一年内，本临床研究应在本院启动。逾期未启动的，本批件自行废止。		
年度/定期跟踪审查 Continue Review	审查频率为该研究批准之日起每 12 月一次，<br>首次，请于 2025 年 8 月 1 日前 1 个月递交研究进展报告。<br>伦理审查委员会有根据实际进展情况改变跟踪审查频率的权利。		
声明 Statement	本伦理审查委员会的职责、人员组成、操作程序及记录遵循《涉及人的生物医学研究伦理审查办法》、《涉及人的生命科学和医学研究伦理审查办法》、《涉及人的健康相关研究国际伦理准则》、《赫尔辛基宣言》、GCP 和 ICH-GCP 等国际伦理指南和国内相关法律法规。		

注意事项：

1. 请遵循我国相关法律、法规和规章中的伦理原则。
2. 请遵循经本伦理审查委员会批准的临床研究方案、知情同意书、招募材料等开展本研究，保护研究参与者的健康与权利。对研究方案、知情同意书和招募材料等的任何修改，均须得到本伦理审查委员会审查同意后方可实施。
3. 在本院发生的SAE/SUSAR以及研发期间安全性更新报告须按照NMPA/GCP最新要求及时递交本伦理审查委员会，国内外其它中心发生的SAE/SUSAR需定期汇总、评估后递交本伦理审查委员会。
4. 根据报告情况，本伦理审查委员会有权对其评估做出新的决定。
5. 自今日起，无论研究开始与否，请在跟踪审查日到期前1个月提交研究进展报告。
6. 申办方应当向组长单位伦理审查委员会提交中心研究进展报告汇总；当出现任何可能显著影响研究进行或增加研究参与者危险的情况时，请申请人及时向本伦理审查委员会提交书面报告。
7. 研究纳入了不符合纳入标准或符合排除标准的研究参与者，符合中止研究规定而未让研究参与者退出研究，给予错误治疗或剂量，给予方案禁止的合并用药等没有遵从方案开展研究的情况；或可能对研究参与者的权益或健康以及研究的科学性造成不良影响等违背GCP原则的情况，请申办方、监查员或研究者提交违背方案报告。
8. 申请人暂停或提前终止临床研究，请及时提交暂停或终止研究报告。
9. 完成临床研究，请申请人提交结题报告。
10. 采集、保藏、利用、对外提供我国人类遗传资源，应当遵守中华人民共和国人类遗传资源管理条例。
11. 凡经本伦理审查委员会批准的研究项目在实施前，申请人应按相关规定在国家卫健委、药审中心等的临床研究登记备案信息系统平台登记研究项目相关信息。

The Medical Ethics Committee Scientific Research Approval Document of People's Hospital of Anji

Ethics Approval Number: (P20230902-1)

Project Title: Clinical Study on the Effect of Early Life and Medical Interventions on Reducing the Incidence and Severity of Carbon Monoxide Poisoning

Project Source: Huzhou Municipal Science and Technology Bureau

Project Leader: Weiguang Wang
Applying Department: Emergency Department

Review Category: Scientific Research Project
Review Method: Meeting Review

Review Date: 20230902
Review Location: People's Hospital of Anji

List of Review Documents:

1.Initial Review Application Form (Version Number: 001, Version Date: 20230902)
2.Research Protocol (Version Number: 001, Version Date: 20230902)
3.Informed Consent Form (Version Number: 001, Version Date: 20230902)
4.Researcher's Curriculum Vitae

Review Committee Members:
Meiqin Dai, Xiaozheng Wang, Chen Xiutang, Jianfei Huang, Guyan Ling, Wenjuan Cao, Lina Wang, Yunchao Guo, Hongbiao Chen.

Review Opinion:
After review by this ethics committee, the scientific research is approved for implementation.
This scientific research will be subject to follow-up review by the ethics committee: ☑ Yes □ No
The frequency of review is: □ 3 months □ 6 months ☑ 12 months from the date of approval.
The ethics committee reserves the right to change the frequency of follow-up reviews based on actual progress.

Chairperson's Signature: Xiaozheng Wang
Date: 2023.9.2

Note: (Please read carefully)
Please adhere to the ethical principles outlined in relevant domestic and international laws and regulations, including the "Administrative Measures for Clinical Research Projects in Healthcare Institutions," "Ethical Review Measures for Biomedical Research Involving

Human Subjects," the WMA (Declaration of Helsinki), and the CIOMS "International Ethical Guidelines for Biomedical Research Involving Human Subjects."

1.This approval document is valid for one year. Whether the research has commenced or not, please submit follow-up review materials one month before the follow-up review date. The research may only continue after obtaining the ethics committee's approval for an extension of the approval document's validity period.
2.Please conduct the scientific research in accordance with the protocol approved by this ethics committee. Any modifications to the research protocol, informed consent form, recruitment materials, or changes in the project leader must be reviewed and approved by the ethics committee before implementation.
3.Any serious adverse events occurring in this hospital or unexpected events affecting the risk-benefit ratio of the research must be promptly reported to this ethics committee.
4.Any deviations from the trial protocol must be promptly reported to this ethics committee.
5.If the research is suspended or terminated prematurely, a suspension or termination report should be submitted promptly. Upon completion of the research, please submit a research completion report for review by this ethics committee.

Address: 699 Tianmu Middle Road, Anji County, Zip Code: 313300
Phone: 5034555 ext. 8552
Contact Person: Wenjuan Cao
Email: ajxrmyyl1@163.com

Department: The Medical Ethics Committee of People's Hospital of Anji
File Number: AF-SC-08(1.0)
File Name: Ethics Committee Approval Letter of Biomedical Research Involving Humans
Version Number: 1.0 **Page Number:** 1/2

Ethics Committee Approval Letter of Biomedical Research Involving Humans

Approval NO: P20230902-1-2
Study Title: Clinical Study on the Effect of Early Life and Medical Interventions on Reducing the Incidence and Severity of Carbon Monoxide Poisoning
Sponsor/Funder: Huzhou Municipal Science and Technology Bureau
Acceptance Number: P20230902-1
Principal Investigator: Weiguang Wang. **Responsible Department:** Emergency Department
Category of Review: Research Project. **Type of Review:** Expedited Review
Date of Review: 20240801 **Location of Review:** Ethics Committee Office
Items Reviewed: Research Progress Report
Evaluation: Approved
Decision: The committee's decision on this project is: ☑ Approval
Chair Signature: Xiaozheng Wang
Date of Issue: 2024.8.1
Stamp of EC: Ethics Committee Seal
Contact Information: Phone: 0572-5665621 Email: ajxrmyy@163.com
Address: No. 699, Tianhuangping Middle Road, Changshuo Street, Anji County, Huzhou City, Zhejiang Province。
Period of Validity: From the date of initial approval by this ethics committee, the clinical research should be initiated in this hospital. If not initiated within the deadline, this approval will be automatically invalidated.
Continue Review: The review frequency is once every 12 months from the date of approval. The first report should be submitted one month before August 1, 2025.The Ethics Committee has the right to change the review frequency according to the actual progress.
Statement: Declaration: The responsibilities, personnel composition, operating procedures, and records of The Ethics Committee comply with the "Regulations on Ethical Review of Biomedical Research Involving Humans," "Regulations on Ethical Review of Life Sciences and Medical Research Involving Humans," "International Ethical Guidelines for Health-Related Research Involving Humans," the Declaration of Helsinki, GCP, and ICH-GCP, as well as other relevant domestic laws and regulations.

Notice:

1. Please follow the ethical principles in the relevant laws, regulations, and rules of our country.
2. Please conduct this research in accordance with the clinical research plan, informed consent form, recruitment materials, etc., approved by The Ethics Committee, and protect the health and rights of research participants. Any modifications to the research plan, informed consent form, and recruitment materials must be reviewed and approved by The Ethics Committee before implementation.
3. Any SAE/SUSAR occurring in this hospital and safety updates during the research and development period must be submitted to The Ethics Committee in a timely manner according to the latest requirements of NMPA/GCP. SAE/SUSAR occurring in other domestic and foreign centers need to be regularly summarized and evaluated before being submitted to The Ethics Committee.
4. Based on the report, The Ethics Committee has the right to make new decisions on its assessment.
5. From today onwards, regardless of whether the research has started, please submit a research progress report one month before the tracking review date expires.
6. The sponsor should submit a summary of the research progress report to the leading unit's ethics committee; when any situation that may significantly affect the progress of the research or increase the risk to participants occurs, the applicant should submit a written report toThe Ethics committee in a timely manner.
7. If the research includes participants who do not meet the inclusion criteria or meet the exclusion criteria, participants who should have been withdrawn from the study according to the regulations but were not, participants who received incorrect treatment or dosage, or participants who received prohibited combined medications that did not comply with the research plan; or if there is any situation that may adversely affect the rights or health of the participants and the scientific nature of the research, violating GCP principles, the sponsor, monitor, or researcher should submit a non-compliance report.
8. If the applicant suspends or terminates the clinical research, please submit a suspension or termination report in a timely manner.
9. Upon completion of the clinical research, the applicant should submit a final report.
10. The collection, preservation, use, and provision of human genetic resources in our country should comply with the Regulations on the Administration of Human Genetic Resources of the People's Republic of China.
11. Before the implementation of any research project approved by The Ethics Committee, the applicant should register the relevant information of the research project on the clinical research registration and filing information system platform of

the National Health Commission, Drug Review Center, etc., in accordance with relevant regulations.
