# Supplementary material for: Global patterns and trends of carbon monoxide poisoning: A comprehensive spatiotemporal analysis using joinpoint regression and ARIMA modeling, 1990–2021
Source: PLoS One. 2025 Aug 22;20(8):e0330778. doi: 10.1371/journal.pone.0330778 (PMC12373207; doi:10.1371/journal.pone.0330778)
Supplement: Attachment — Submitted filename: Review report Plos one.docx [file pone.0330778.s002.docx]

Ehsan Hashim Abu-Zeid	19^th^ April 2025

Forensic Medicine and Toxicology Depart.,

Faculty of Veterinary Medicine,

Zagazig University, Egypt

**Dear respected Editor of PLOS ONE Journal,**

Many thanks for your confidence for peer reviewing of the original research article entitled “**Global Patterns and Trends of Carbon Monoxide Poisoning: A Systematic Analysis from 1990 to 2021”** with manuscript number: **PONE-D-25-08215** for consideration in **PLOS ONE.**

The study seems apparently well, I enjoyed reading it. Yet, few concerns are required before for publication.

**Major concerns**

1. Title is concise and should contain the name of the main used analytical methods or the limitations solving
2. The abstract needs to be reorganized firstly to clarify the main aim of your study to overcome the limitations of the previous studies in the same line with your research.
3. What are the validity of the used analytical methods main advantages and disadvantages and why such methods were applied (joinpoint regression, spatial statistics, and ARIMA modeling).
4. Abstract can’t stand alone. All results were mentioned in very concise manner that not clear for authors and the conclusive words not satisfactory about your obtained results versus your study aim.
5. Keywords not representative for your study aim or methods or analysis
6. More reviewing data about the CO poisoning are required in the section of introduction. What is the main health problems reported from exposure to CO and how exposure occur?
7. Line 63 lesions not satisfactory for the side effects of HFM replace with renal damage or renal toxicity from pesticide exposure.
8. Material and methods section: ethical approval code must be supplied in such section not at the end of the manuscript
9. Not all the obtained data were fully discussed. Discussion are very concise
10. Conclusion is so concise not presenting the study results or significance
11. All the abbreviations must be mentioned in full name for the first time and list of abbreviations should be supplied
12. Grammatical errors, has major grammatical and structural errors. Please, double-check. English must be improved and certified.

Sincerely,

Ehsan
