# Supplementary material for: Global patterns and trends of carbon monoxide poisoning: A comprehensive spatiotemporal analysis using joinpoint regression and ARIMA modeling, 1990–2021
Source: PLoS One. 2025 Aug 22;20(8):e0330778. doi: 10.1371/journal.pone.0330778 (PMC12373207; doi:10.1371/journal.pone.0330778)
Supplement: Attachment — Submitted filename: Global Patterns and Trends of Carbon Monoxide Poisoning.docx [file pone.0330778.s004.docx]

### ****Manuscript Title:****

Global Patterns and Trends of Carbon Monoxide Poisoning: A Systematic Analysis from 1990 to 2021

### ****Recommendation: Minor Revision****

### ****General Comments****

This manuscript presents a comprehensive and methodologically rigorous analysis of the global burden of acute carbon monoxide (CO) poisoning using data from the GBD 2021 study. The **Discussion section** is particularly well-developed, integrating key findings with socio-demographic insights and regional disparities. It adds value by addressing gender differences, projecting future trends, and offering policy implications.

The paper is timely and relevant for global health policy, environmental epidemiology, and injury prevention fields.

### ****Strengths****

- Clear articulation of global trends in CO burden (incidence, mortality, DALYs).
- Integration of **socio-demographic index (SDI)** as a framework enhances interpretability.
- Thoughtful consideration of **gender-specific trends** and **regional disparities**.
- Well-grounded policy recommendations and recognition of **forecast uncertainty**.
- Transparent acknowledgement of **data limitations** and methodological constraints.

### ****Minor Revisions Requested****

**Reduce Repetition of Quantitative Results**

The Discussion section occasionally reiterates specific incidence and mortality figures already presented in the Results. Consider summarizing trends without repeating exact numbers.

**Expand Policy Examples**

Strengthen the applicability of policy suggestions by referencing **concrete measures** (e.g., mandatory CO detectors in homes, national surveillance programs in Eastern Europe or Canada).

**Consider Behavioral and Climate Factors**

Briefly address how **climate change** and **seasonal factors** may affect CO poisoning trends.

Include a sentence on the potential impact of **behavioral interventions** (e.g., public education, alarm usage).

**Clarify Forecasting Limitations**

While limitations are mentioned, highlight the **potential variability** due to emerging technologies or policy shifts (e.g., clean energy transitions, urbanization).

### ****Conclusion****

This is a strong manuscript with high public health relevance and rich global insights. Minor editorial and content refinements will further improve clarity and impact. Once revised, it will make a valuable contribution to the literature on environmental health and injury epidemiology.
