# Supplementary material for: Global patterns and trends of carbon monoxide poisoning: A comprehensive spatiotemporal analysis using joinpoint regression and ARIMA modeling, 1990–2021
Source: PLoS One. 2025 Aug 22;20(8):e0330778. doi: 10.1371/journal.pone.0330778 (PMC12373207; doi:10.1371/journal.pone.0330778)
Supplement: Attachment — Submitted filename: Response to Reviewers.docx [file pone.0330778.s006.docx]

Response to Reviewers

Manuscript ID: PONE-D-25-08215

Title: Global Patterns and Trends of Carbon Monoxide Poisoning: A Systematic Analysis from 1990 to 2021

Dear Dr. Antonio Peña-Fernández and Esteemed Reviewers,

We are deeply grateful for the thorough and constructive feedback provided during the peer review process. The insightful comments have significantly improved the quality and clarity of our manuscript. We have carefully addressed each concern and suggestion, and we believe the revised manuscript is substantially strengthened as a result.

Response to Editorial Requirements

Dear Dr. Peña-Fernández,

We sincerely thank you for your editorial guidance and the opportunity to revise our manuscript. We greatly appreciate your patience and the comprehensive review process you have facilitated.

Editorial Requirement 1: PLOS ONE Style Requirements

**Response:**

We have carefully reviewed and implemented all PLOS ONE formatting requirements, including file naming conventions and manuscript structure. We have consulted both provided style templates to ensure full compliance. We have used red font to highlight all modifications made throughout the manuscript in response to the reviewers' feedback.

Editorial Requirement 2: Data Availability Statement

**Response:**

We fully understand and value the importance of data transparency. This study uses publicly available data from the Global Burden of Disease Study 2021 database (accessible at: http://ghdx.healthdata.org/), and all supporting data are openly available without restrictions. We have prepared supplementary files containing the processed data versions used to create all figures. These files, named "minimal data set.rar", will be uploaded along with the revised version as supporting information files.

Editorial Requirement 3: Ethics Statement Placement

**Response:**

Thank you for this important clarification regarding the placement of the ethics statement. We have revised the manuscript accordingly:

1. Moved the ethics statement to the Methods section: We have consolidated and moved the complete ethics statement to the "Study Design and Data Sources" subsection of the Methods section, ensuring all ethical considerations are properly documented in the appropriate location.
2. Removed the standalone Ethics Statement section: The separate ethics statement that previously appeared at the end of the manuscript has been deleted to avoid duplication.
3. Enhanced the ethics content in Methods: The ethics statement in the Methods section now includes comprehensive information about ethical approval, informed consent waiver justification, and compliance with relevant ethical guidelines.

The ethics statement now appears only in the Methods section as required and contains all necessary ethical information for publication.

We conducted a comprehensive analysis of acute carbon monoxide poisoning using the Global Burden of Disease (GBD) 2021 database, accessed through the Global Health Data Exchange (GHDx) platform[9]. The analysis covered the period from 1990 to 2021, encompassing data from 204 countries and territories. Our research framework adhered to the GATHER guidelines for health estimates reporting[10]. The data were accessed for research purposes on March 15, 2021.

This study was conducted in accordance with the ethical standards of the institutional and/or national research committee and with the 1964 Helsinki Declaration and its later amendments or comparable ethical standards. The need for informed consent was waived by the ethics committee because the study involved the analysis of fully anonymized retrospective data from the Global Burden of Disease Study 2021. All data were accessed after complete anonymization, and no individual identifiers were included in the analysis. All analyses were conducted in compliance with ethical guidelines for human subjects research.

Editorial Requirement 4: Map Copyright Issues

**Response:**

Thank you for raising this important copyright concern regarding the map images in our submission. We appreciate your diligence in ensuring compliance with CC BY 4.0 license requirements. Please find our detailed response below:

Map Creation and Data Sources:

The map images in Figures 1, 2, and 3 were created entirely using public domain resources and open-source tools:

Base map data: Natural Earth (https://www.naturalearthdata.com/), accessed via the rnaturalearth R package (https://github.com/ropensci/rnaturalearth)

Disease burden data: Global Burden of Disease Study 2021 (public database)

Creation software: R programming language with ggplot2 and sf packages (open-source)

Copyright and License Compatibility:

Natural Earth explicitly releases all their map data into the public domain with no copyright restrictions. According to Natural Earth's terms of use (https://www.naturalearthdata.com/about/terms-of-use/), their data can be used for any purpose without permission, making it fully compatible with PLOS ONE's CC BY 4.0 license requirements. No proprietary software (such as Google Maps, ArcGIS, or other commercial mapping platforms) was used in creating these figures.

Updated Figure Captions with Proper Attribution:

We have revised the figure captions to include appropriate attribution:

Figure 1: Geographic Distribution of Age-Standardized Incidence Rates of Acute Carbon Monoxide Poisoning (per 100,000 population) in 2021. Disease burden data from Global Burden of Disease Study 2021; Map data from Natural Earth (https://www.naturalearthdata.com/), public domain.

Figure 2: Geographic Distribution of Age-Standardized Mortality Rates of Acute Carbon Monoxide Poisoning (per 100,000 population) in 2021. Disease burden data from Global Burden of Disease Study 2021; Map data from Natural Earth (https://www.naturalearthdata.com/), public domain.

Figure 3: Geographic Distribution of Age-Standardized DALY Rates of Acute Carbon Monoxide Poisoning (per 100,000 population) in 2021. Disease burden data from Global Burden of Disease Study 2021; Map data from Natural Earth (https://www.naturalearthdata.com/), public domain.

Compliance Confirmation:

All map figures in our submission are created using public domain data and open-source software, ensuring full compliance with CC BY 4.0 licensing requirements. These figures can be freely accessed, downloaded, copied, distributed, and used by any third party, including for commercial purposes, with proper attribution as specified in the updated captions.

We believe this addresses all copyright concerns while maintaining the scientific integrity and visual clarity of our geographical analysis.

**Response to Reviewer #1**

We extend our heartfelt gratitude to Reviewer #1 for the comprehensive and constructive feedback. Your detailed suggestions have significantly enhanced the scientific rigor and clarity of our manuscript. We have addressed each point systematically:

**Comment 1:**

**Reviewer's Suggestion:** "Title is concise and should contain the name of the main used analytical methods or the limitations solving."

**Our Response:**

We deeply appreciate this valuable suggestion. We have revised the title to better reflect our methodological contributions:

Original Title: "Global Patterns and Trends of Carbon Monoxide Poisoning: A Systematic Analysis from 1990 to 2021"

Revised Title: "Global Patterns and Trends of Carbon Monoxide Poisoning: A Comprehensive Spatiotemporal Analysis Using Joinpoint Regression and ARIMA Modeling, 1990-2021"

This revision explicitly highlights our key analytical methods (joinpoint regression, ARIMA modeling) and emphasizes the spatiotemporal nature of our analysis.

**Comment 2:**

**Reviewer's Suggestion:** "The abstract needs to be reorganized firstly to clarify the main aim of your study to overcome the limitations of the previous studies."

**Our Response:** Thank you for this crucial insight. We have completely restructured our abstract to explicitly address how our study overcomes previous research limitations:

**Abstract**

**Background:** Carbon monoxide (CO) poisoning causes approximately 41,000 deaths annually worldwide despite being preventable. Previous studies focused primarily on mortality alone, lacked systematic socio-demographic analysis, and provided no predictive models. This study comprehensively analyzes global CO poisoning patterns using spatiotemporal methods to inform evidence-based prevention strategies.

**Methods:** We analyzed Global Burden of Disease Study 2021 data from 204 countries (1990-2021) for age-standardized incidence, mortality, and disability-adjusted life years (DALYs). Joinpoint regression identified temporal trends with statistical precision, spatial statistics quantified geographic clustering, and ARIMA modeling projected trends through 2050. We examined associations with socio-demographic index (SDI) across regions and countries.

**Results:** Global age-standardized incidence rates decreased significantly by 35.1% from 12.13 (95% UI: 8.30-17.00) to 7.87 (95% UI: 5.54-10.81) per 100,000 population (annual percentage change: -1.16%, 95% UI: -1.35% to -0.96%, p<0.001). Mortality rates declined more dramatically by 53.9% from 0.76 (95% UI: 0.66-0.91) to 0.35 (95% UI: 0.24-0.40) per 100,000 (annual change: -2.79%, 95% UI: -3.14% to -2.44%, p<0.001). DALY rates showed the steepest reduction of 59.5% from 37.59 (95% UI: 31.75-44.76) to 15.22 (95% UI: 10.67-17.57) per 100,000 (annual change: -3.18%, 95% UI: -3.51% to -2.84%, p<0.001). Eastern Europe demonstrated the highest burden (37.98 per 100,000 in 2021). Males experienced significantly higher mortality than females (0.50 vs 0.20 per 100,000, p<0.001). SDI analysis revealed an inverted U-shaped relationship (Spearman's r=0.76, p<0.001), with peak burden at moderate development levels (SDI: 0.6-0.7).

**Conclusions:** These findings directly address previous research gaps by demonstrating: (1) faster mortality decline than incidence decline indicates improved global treatment capabilities; (2) the SDI-burden relationship identifies moderate-development countries as priority intervention targets; (3) significant male predominance (2.5-fold higher mortality) supports gender-specific prevention programs; and (4) persistent Eastern European hotspots require targeted infrastructure improvements. Predictive models forecast continued decline through 2050 and enable evidence-based healthcare planning. This comprehensive analysis provides the first multi-dimensional global assessment, offering crucial evidence for differentiated prevention strategies worldwide.

**Comment 3:**

**Reviewer's Suggestion:** "What are the validity of the used analytical methods main advantages and disadvantages and why such methods were applied."

**Our Response:**

Thank you for this important methodological inquiry. We selected three complementary analytical approaches, each with specific advantages, limitations, and applications. Here is our detailed justification:

1. Joinpoint Regression Analysis

Advantages:

Objectively identifies significant change points in temporal trends without a priori assumptions

More accurately captures complex, non-linear temporal patterns compared to traditional linear regression

Provides statistically precise estimates of Annual Percentage Change (APC) and Average Annual Percentage Change (AAPC)

Well-established methodology widely used in disease surveillance and epidemiological research

Limitations:

Requires sufficient data points (typically ≥4 time points) for reliable results

Sensitive to outliers in the data

May overfit when data are limited

Rationale for Use: With 31 years of continuous data (1990-2021), joinpoint regression was optimal for identifying long-term trend changes in CO poisoning burden, particularly detecting temporal impacts of policy interventions or socioeconomic changes on disease patterns.

2. Spatial Statistics Analysis

Advantages:

Identifies spatial clustering and hotspot regions of disease burden

Accounts for geographic proximity effects on disease distribution

Global Moran's I and LISA analyses provide quantitative assessment of global and local spatial autocorrelation

Facilitates identification of priority geographic areas for intervention

Limitations:

Requires high-quality geocoded data

May be affected by administrative boundary effects

Choice of spatial weight matrix can influence results

Rationale for Use: CO poisoning is often closely related to geographic factors (climate, heating methods, infrastructure). Spatial statistical analysis reveals these geographic patterns, providing scientific evidence for developing region-specific prevention strategies.

3. ARIMA Time Series Modeling

Advantages:

Handles autocorrelation and seasonality in time series data

Provides scientific predictions based on historical data patterns

Offers uncertainty intervals for prediction results

Widely applied and reliable methodology in epidemiological forecasting

Limitations:

Assumes historical trends will continue into the future, potentially missing impacts of sudden events

Cannot directly incorporate external covariates (policy changes, technological advances)

Long-term prediction uncertainty increases progressively

Rationale for Use: To support long-term health planning and resource allocation, we needed to forecast future trends in CO poisoning burden. ARIMA modeling, based on 31 years of historical data, provides reasonable projections through 2050, offering prospective information for policymakers.

Methodological Integration Rationale:

These three methods are complementary and form a comprehensive analytical framework: joinpoint regression describes historical trends, spatial statistics reveals geographic patterns, and ARIMA modeling predicts future trends. This multi-method integration provides a complete assessment of the global burden of CO poisoning.

Validity Considerations:

All methods were validated through appropriate statistical tests, model diagnostics, and uncertainty quantification. The combination approach enhances the robustness of our findings and provides multiple perspectives on the same epidemiological phenomenon.

**Comment 4:**

**Reviewer's Suggestion:** "Abstract can't stand alone. All results were mentioned in very concise manner that not clear for authors and the conclusive words not satisfactory."

**Our Response:** We are grateful for this observation. We have substantially enhanced the abstract to ensure it stands alone effectively.

**Comment 5:**

**Reviewer's Suggestion:** "Keywords not representative for your study aim or methods or analysis."

**Our Response:** Thank you for this important feedback. We have revised our keywords to better reflect our study's methodological and analytical focus:

Revised Keywords: carbon monoxide poisoning; joinpoint regression; ARIMA modeling; spatiotemporal analysis; socio-demographic index; global burden of disease; health disparities; forecasting

**Comment 6:**

**Reviewer's Suggestion:** "More reviewing data about the CO poisoning are required in the section of introduction. What is the main health problems reported from exposure to CO and how exposure occur?"

**Our Response:** We deeply appreciate this suggestion for strengthening our literature foundation. We have added a comprehensive paragraph detailing:

Carbon monoxide exposure occurs through multiple pathways. Common sources include faulty heating systems, poorly ventilated cooking appliances, vehicle exhaust in enclosed spaces, and fuel-burning equipment such as generators[4, 5]. Motor vehicle exhaust represents a significant source of CO exposure, particularly from stationary vehicles in enclosed spaces[6]. The health impacts range from acute symptoms including headache, dizziness, and nausea at concentrations of 50-100 ppm to severe neurological damage, cardiac arrhythmias, and death at concentrations exceeding 400 ppm[7]. Long-term sequelae among survivors include persistent neurological deficits, cognitive impairment, and increased risk of delayed neurological sequelae affecting 10-32% of patients[7].

**Comment 7:**

**Reviewer's Suggestion:** "Line 63 lesions not satisfactory for the side effects of HFM replace with renal damage or renal toxicity from pesticide exposure."

**Our Response:** Thank you for your feedback. However, we believe there may be some confusion regarding your comment on Line 63. Our manuscript focuses on the global burden of carbon monoxide poisoning and does not discuss HFM side effects or pesticide-related renal toxicity.

**Comment 8:**

**Reviewer's Suggestion:** "Material and methods section: ethical approval code must be supplied in such section not at the end of the manuscript."

**Our Response:**

Thank you for this important procedural guidance. We have moved the ethics statement to the Methods section with the following clarification:

We conducted a comprehensive analysis of acute carbon monoxide poisoning using the Global Burden of Disease (GBD) 2021 database, accessed through the Global Health Data Exchange (GHDx) platform[13]. The analysis covered the period from 1990 to 2021, encompassing data from 204 countries and territories. Our research framework adhered to the GATHER guidelines for health estimates reporting[14]. The data were accessed for research purposes on March 15, 2021.

This study was conducted in accordance with the ethical standards of the institutional and/or national research committee and with the 1964 Helsinki Declaration and its later amendments or comparable ethical standards. The need for informed consent was waived by the ethics committee because the study involved the analysis of fully anonymized retrospective data from the Global Burden of Disease Study 2021. All data were accessed after complete anonymization, and no individual identifiers were included in the analysis. All analyses were conducted in compliance with ethical guidelines for human subjects research.

**Comment 9:**

**Reviewer's Suggestion:** "Not all the obtained data were fully discussed. Discussion are very concise."

**Our Response:**

Thank you for pointing out that our discussion could be more comprehensive. We have expanded the discussion section by adding a detailed paragraph that thoroughly addresses several key findings that were not fully explored in the original version. We believe this addition significantly strengthens the discussion by providing deeper insights into our comprehensive dataset and better connecting our findings to actionable public health implications.

Several novel findings from our comprehensive analysis warrant detailed discussion. The differential decline rates between incidence (-1.16% annually) and mortality (-2.79% annually) suggest that while exposure prevention efforts are moderately successful, treatment improvements have been more substantial, particularly in high-SDI regions. This pattern indicates that healthcare capacity building may be more achievable than primary prevention in the short term[29]. The inverted U-shaped SDI relationship reveals a critical insight: countries at moderate development levels (SDI 0.6-0.7) face the highest burden, likely reflecting increased fossil fuel use during industrialization without adequate safety infrastructure. This "development trap" suggests that economic growth alone does not guarantee reduced CO poisoning risk without targeted interventions[30]. The pronounced male predominance (2.5-fold higher mortality) extends beyond occupational exposure to include behavioral factors such as risk-taking behaviors and delayed healthcare seeking[31]. Our geographic clustering analysis reveals that neighboring countries often share similar burden patterns regardless of SDI differences, highlighting the importance of regional cooperation in prevention strategies. The persistent Eastern European hotspot, despite three decades of observation, underscores the complex interplay between climate, infrastructure legacy, and socioeconomic transition that requires sustained, multifaceted interventions.

**Comment 10:**

**Reviewer's Suggestion:** "Conclusion is so concise not presenting the study results or significance."

**Our Response:** We are grateful for this feedback. We have completely rewritten and substantially expanded our Conclusions section to comprehensively present:

Conclusion

This comprehensive global analysis of carbon monoxide poisoning from 1990-2021 reveals significant epidemiological patterns with important public health implications. Our study demonstrates a substantial global decline in CO poisoning burden, with age-standardized incidence rates decreasing by 35.1% from 12.13 to 7.87 per 100,000 population, mortality rates declining by 2.79% annually, and DALYs showing the steepest reduction at 3.18% annually. Despite these improvements, nearly 300,000 new cases and 29,000 deaths occurred globally in 2021, indicating continued substantial burden.

Critical disparities persist across regions and demographics. Eastern Europe maintains the highest burden with incidence rates of 37.98 per 100,000—five times the global average. Males experience 2.5 times higher mortality rates than females across all regions. Most significantly, our analysis reveals a novel inverted U-shaped relationship between socio-demographic development and disease burden, with countries at moderate SDI levels (0.6-0.7) experiencing peak mortality and DALYs burden, while high-SDI regions show higher incidence but dramatically lower case-fatality rates (1.24% vs. 4.26%).

These findings have profound public health significance, demonstrating that economic development alone does not guarantee reduced CO poisoning burden without targeted interventions. The data support differentiated prevention strategies: infrastructure modernization in high-burden regions, strengthened medical capacity in transitioning economies, and enhanced surveillance in low-resource settings. Our projections through 2050 indicate continued global decline but persistent regional disparities, emphasizing the need for sustained, equity-focused interventions. This study provides the first multi-dimensional global assessment of CO poisoning burden and establishes a framework for evidence-based policy making to address this entirely preventable cause of death and disability worldwide.

**Comment 11:**

**Reviewer's Suggestion:** "All the abbreviations must be mentioned in full name for the first time and list of abbreviations should be supplied."

Our Response: Thank you for this essential requirement. We have created a comprehensive abbreviations list and ensured all abbreviations are properly defined at first use:

List of Abbreviations:

APC: Annual Percentage Change

AAPC: Average Annual Percentage Change

ARIMA: Autoregressive Integrated Moving Average

ASR: Age-Standardized Rate

CO: Carbon monoxide

DALY: Disability-Adjusted Life Year

GBD: Global Burden of Disease

GHDx: Global Health Data Exchange

LISA: Local Indicators of Spatial Association

SDI: Socio-Demographic Index

UI: Uncertainty Interval

WHO: World Health Organization

YLD: Years Lived with Disability

YLL: Years of Life Lost

**Comment 12:**

**Reviewer's Suggestion:** "Grammatical errors, has major grammatical and structural errors. Please, double-check. English must be improved and certified."

**Our Response:** We deeply appreciate your attention to language quality. We have conducted comprehensive language editing throughout the manuscript, with particular focus on:

Grammar correction and sentence structure improvement

Enhanced clarity and academic tone

Proper use of articles and prepositions

Consistent tense usage and formal academic expression

Professional English editing consultation

**Response to Reviewer #2**

We are profoundly grateful to Reviewer #2 for the positive evaluation and constructive suggestions. Your recognition of our work's strengths is encouraging, and your recommendations for minor revisions have further enhanced the manuscript's impact.

General Comments Appreciation

We deeply appreciate your recognition that our manuscript presents "a comprehensive and methodologically rigorous analysis" and your acknowledgment of the "particularly well-developed Discussion section." Your positive assessment of our integration of socio-demographic insights and policy implications is especially gratifying.

**Comment 1:**

**Reviewer's Suggestion:** "The Discussion section occasionally reiterates specific incidence and mortality figures already presented in the Results. Consider summarizing trends without repeating exact numbers."

**Our Response:** Thank you for this excellent editorial guidance. We have carefully revised the Discussion section to eliminate numerical repetition while maintaining analytical depth:

Original: "First, our study demonstrates that the global age-standardized incidence rate of CO poisoning decreased from 12.13 per 100,000 in 1990 to 7.87 per 100,000 in 2021..."

Revised: " First, our study demonstrates a substantial global decline in CO poisoning incidence over the study period, reflecting[19], enhanced ventilation facilities[20], and increased public awareness[21]. "

We have applied this approach throughout the Discussion, focusing on trend interpretation rather than data repetition.

**Comment 2:**

**Reviewer's Suggestion:** "Strengthen the applicability of policy suggestions by referencing concrete measures (e.g., mandatory CO detectors in homes, national surveillance programs in Eastern Europe or Canada)."

**Our Response:** We sincerely appreciate this practical suggestion for enhancing policy relevance. We have added a comprehensive paragraph with specific evidence-based interventions:

"These findings support specific evidence-based interventions: mandatory CO detector installation in residential buildings (successful in Canada and United States)[31], national surveillance programs (established in Norway and United Kingdom)[4, 32], government-subsidized heating system replacement programs (implemented in Poland and Czech Republic)[33], and strengthened poison control centers following WHO models in developing countries[34, 35]."

**Comment 3:**

**Reviewer's Suggestion:** "Briefly address how climate change and seasonal factors may affect CO poisoning trends. Include a sentence on the potential impact of behavioral interventions."

**Our Response:** Thank you for highlighting these important environmental and behavioral dimensions. We have added a new section addressing:

"Climate factors significantly influence CO poisoning patterns, with colder temperatures increasing heating demands and associated risks[26, 27]. Climate change may alter geographic risk patterns, potentially reducing cold-weather exposures while creating new energy-related risks[28]. Behavioral interventions, including public education campaigns, proper equipment maintenance awareness, and widespread CO detector adoption, have proven effective in reducing mortality trends in developed nations[29, 30]."

**Comment 4:**

**Reviewer's Suggestion:** "While limitations are mentioned, highlight the potential variability due to emerging technologies or policy shifts (e.g., clean energy transitions, urbanization)."

**Our Response:** We deeply value this forward-looking perspective. We have expanded our limitations section to address emerging uncertainties:

"Additionally, our forecasting models may not fully capture emerging variables that could significantly alter future trends. The global transition to clean energy and electric heating systems may accelerate burden decline beyond our projections. Conversely, rapid urbanization, technological innovations in smart monitoring systems, carbon neutrality policies, and climate change-induced extreme weather events introduce variability not reflected in historical trend-based models. These factors suggest our projections represent baseline scenarios rather than definitive forecasts."

Final Comments Appreciation

We are deeply honored by your conclusion that this is "a strong manuscript with high public health relevance and rich global insights" and your assessment that it "will make a valuable contribution to the literature on environmental health and injury epidemiology."

Summary of Major Revisions

In response to all reviewers' valuable feedback, we have implemented the following substantial improvements:

1.Enhanced methodological transparency with detailed analytical rationale

2.Comprehensive abstract restructuring addressing previous research gaps

3.Substantially expanded Discussion and Conclusions sections

4.Addition of specific policy recommendations with concrete examples

5.Integration of climate and behavioral factors analysis

6.Enhanced forecasting limitations discussion with emerging factors

7.Complete language editing and grammar improvement

8.Proper abbreviations list and formatting compliance

9.Comprehensive ethics and data availability clarifications

10.Copyright compliance verification and proper attribution

We believe these revisions have transformed our manuscript into a significantly stronger contribution to the literature. The enhanced methodological rigor, expanded policy relevance, and improved clarity directly address all reviewer concerns while maintaining the scientific integrity of our findings.

We are deeply grateful for the thorough review process and the opportunity to improve our work. The constructive feedback has not only enhanced this manuscript but has also provided valuable learning opportunities for our future research endeavors.

Thank you once again for your time, expertise, and dedication to advancing scientific knowledge in environmental health and injury epidemiology.

With sincere appreciation and respect,

Dr. Weiguang Wang and Co-authors
